# Structural and functional characterisation of a reconstructed ancestral strigolactone receptor

**DOI:** 10.1111/tpj.71113

**Published:** 2026-09-14

**Authors:** Andrew J. Tuckey, Andrew C. Marshall, Emel Rothzerg, Antonella P. Monte, Tom Bennett, Charles S. Bond, Mark T. Waters

**Affiliations:** ^1^ School of Molecular Sciences The University of Western Australia Perth Western Australia Australia; ^2^ School of Biomedical Sciences The University of Western Australia Perth Western Australia Australia; ^3^ School of Biology University of Leeds Leeds UK

**Keywords:** strigolactone, butenolide, ancestral sequence reconstruction, hormone signalling, *Arabidopsis thaliana*

## Abstract

Strigolactones are phytohormones that regulate shoot branching and facilitate communication with neighbouring plants, bacteria and arbuscular mycorrhizal fungi. In seed plants, strigolactone perception begins with the enzyme‐receptor DWARF14 (D14), an α/β‐hydrolase likely to have evolved via gene duplication within a larger gene family that includes the karrikin receptor, KARRIKIN INSENSITIVE 2 (KAI2) and other D14‐like (DLK) proteins of uncertain function. However, it is unclear when the functional characteristics that define a *bona fide* strigolactone receptor evolved. Here, we apply ancestral sequence reconstruction to generate a D14 protein representative of seed plants to study the evolution of ligand specificity and the conservation of signalling mechanisms and to explore desirable traits for protein engineering. Ancestrally reconstructed D14 is structurally and functionally comparable to D14 from *Arabidopsis thaliana*. The ligand specificity of ancestral D14 does not meaningfully differ, despite having unusual active site geometry and altered enzyme kinetics. We show that the catalytic aspartic acid residue of ancestral D14 is not required for strigolactone signalling when expressed in *A. thaliana*, suggesting that this mechanistic feature is conserved throughout seed plants. We find that ancestral D14 exhibits higher recombinant yields, greatly increased thermostability and enhanced catalytic activity relative to D14 from *A. thaliana*. This work provides insight into the evolution of phytohormone signalling and presents a robust scaffold for the application of D14‐type proteins in diverse applications.

## Significance Statement

Ancestral sequence reconstruction of the strigolactone receptor DWARF14 (D14) reveals that its substrate specificity and signalling mechanism have remained largely conserved in seed plants. Ancestral D14 exhibits improved recombinant yields, thermostability and catalytic activity relative to D14 from *Arabidopsis thaliana*, providing evolutionary insights and utility for synthetic biology applications.

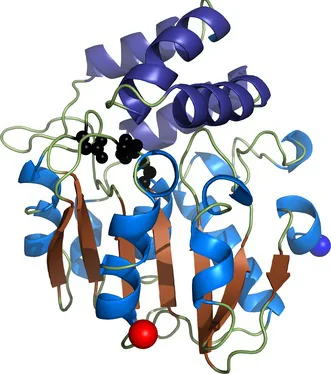

## INTRODUCTION

Strigolactones (SLs) are a class of butenolide phytohormones that regulate numerous processes in land plants. SLs were initially identified as molecules exuded into the soil from plant roots that stimulate the germination of parasitic weeds in the genus *Striga* (Cook et al., 1966). The beneficial role of SLs for the host plant remained enigmatic until it was observed that the phytohormones also promote symbiosis with arbuscular mycorrhizal (AM) fungi (Akiyama et al., 2005) and root bacteria (Foo & Davies, 2011; Liu et al., 2013). SLs additionally control shoot architecture via the inhibition of shoot branching (Gomez‐Roldan et al., 2008; Umehara et al., 2008). More recent evidence suggests that SLs mediate plant growth responses according to interspecific and intraspecific planting density (Wheeldon et al., 2022; Yoneyama et al., 2022). As such, a fuller understanding of SL biology offers a promising avenue for enhancing crop yields (Kelly et al., 2023).

The α/β‐hydrolase DWARF14 (D14) assumes a dual role as both an enzyme and receptor for SLs. D14 binds the SL ligand in a hydrophobic pocket, cleaves the butenolide D‐ring via nucleophilic attack initiated by the catalytic serine residue and undergoes a conformational change in protein structure (de Saint Germain et al., 2016; Hamiaux et al., 2012; Yao et al., 2016). Which of these specific steps are necessary for signal transduction is unresolved. One model posits that the hydrolysis of the D‐ring bridges the catalytic serine and histidine to form a covalently linked intermediate molecule, which is a required conformation for signal transduction (de Saint Germain et al., 2016, Yao et al., 2016). This model has been disputed due to inconsistency between the time taken to hydrolyse SLs being much longer than the downstream effects of D14 signalling (Seto et al., 2019; Zhao et al., 2013). Additionally, a mutant protein lacking the catalytic aspartic acid residue can facilitate signalling in Arabidopsis, despite being unable to hydrolyse SLs (Seto et al., 2019). These findings imply that ligand binding alone is sufficient for signal transduction, with hydrolysis occurring afterwards, but the generality of this mechanism remains to be demonstrated.

Upon interaction with SLs, the conformational shift in D14 facilitates its interaction with protein partners, including MORE AXILLARY GROWTH 2 (MAX2), an F‐box protein that forms part of an SCF (SKP1–CULLIN–F‐BOX) E3 ubiquitin ligase complex (Hamiaux et al., 2012; Shabek et al., 2018; Yao et al., 2016). In such complexes, the F‐box protein specifies protein targets for polyubiquitination, culminating in their subsequent degradation via the 26S proteasome (Woo et al., 2001). The specific targets for D14‐mediated signalling are SUPPRESSOR OF MAX2‐LIKE 6, −7 and −8 (SMXL6/7/8) in *Arabidopsis thaliana* (Soundappan et al., 2015), orthologous to DWARF53 (D53) in *Oryza sativa* (rice) (Jiang et al., 2013; Zhou et al., 2013). Notably, the D14‐MAX2 complex has also been shown to interact with and mediate the proteolytic degradation of paralogous proteins SMAX1 and SMXL2 to facilitate the regulation of hypocotyl elongation (Wang, Xu, et al., 2020) and osmotic stress tolerance (Li et al., 2022).

SMAX1 and SMXL2 are the canonical downstream targets of KARRIKIN INSENSITIVE 2 (KAI2) signalling (Khosla et al., 2020; Soundappan et al., 2015; Stanga et al., 2013, 2016). KAI2 defines a parallel pathway involved in the perception of abiotic butenolides, known as karrikins, that are derived from smoke (Waters et al., 2012). In part because karrikins are far from ubiquitous in the environment, KAI2 signalling is thought to mediate perception and response to an unknown butenolide phytohormone (‘KAI2 ligand’, or KL) that is distinct from SLs (Conn & Nelson, 2016; Waters & Nelson, 2023; Yao et al., 2021). As *KAI2* homologues are found in all land plants and charophyte algae but *D14* homologues are limited to seed plants, it is thought that D14‐mediated signalling evolved from duplication and neofunctionalisation of KAI2, concomitant with diversification of the SMXL family (Bythell‐Douglas et al., 2017; Walker et al., 2019). The two signalling pathways, although similar, are functionally separate; the presence of KAI2 cannot compensate for the absence of D14 and vice versa (Waters et al., 2015).

As homologous α/β‐hydrolases involved in the perception of butenolide phytohormones, KAI2 and D14 show overlapping substrate preferences. Both respond to the synthetic SL *rac*‐GR24, but they have opposing stereoselectivity. Over a range of species, KAI2 prefers the GR24 enantiomer with a 2′*S* D‐ring (GR24^*ent*‐5DS^) over a 2′*R* D‐ring (GR24^5DS^) (Carbonnel et al., 2020; Guercio et al., 2022; Kodama et al., 2022; Meng et al., 2021; Mizuno et al., 2021; Scaffidi et al., 2014; Sun et al., 2020). In contrast, all known natural SLs have stereochemistry comparable to the latter, and these enantiomers are most bioactive through D14 (Flematti et al., 2016; Umehara et al., 2015). Another differing facet of ligand specificity is the presence of the 4′ methyl group in the butenolide moiety. All known non‐synthetic SLs possess a methyl‐substituted carbon at the 4′ position (Nomura et al., 2024). Synthetic SLs without this methyl group (i.e. desmethyl‐SLs) cannot induce D14‐mediated inhibition of bud outgrowth in *Pisum sativum* (Boyer et al., 2012) nor D14‐mediated suppression of hypocotyl elongation in *A. thaliana* (Yao et al., 2021). D14 does not undergo thermal destabilisation in the presence of desmethyl butenolides such as desmethyl GR24 (dGR24), but is highly sensitive to GR24 with the 4′ methyl group (Yao et al., 2021). Conversely, desmethyl butenolides are preferred by KAI2, as indicated by thermal destabilisation, ligand binding and hydrolysis experiments (Kushihara et al., 2025; Tanaka et al., 2026; Yao et al., 2021). In physiological assays such as the inhibition of seedling hypocotyl elongation, dGR24 is uniquely bioactive through KAI2 (Yao et al., 2021). Enigmatically, D14 hydrolyses desmethyl butenolides at a greater rate than methyl‐substituted equivalents (Yao et al., 2021; Yao, Mashiguchi, et al., 2018). However, because desmethyl butenolides are biologically inactive via D14 and do not thermally destabilise D14 (Yao et al., 2021), desmethyl butenolides likely behave as simple enzymatic substrates that are turned over rapidly, but cannot activate D14 to initiate signalling (de Saint Germain et al., 2016).

Characterised D14 orthologues from angiosperms and gymnosperms can be placed in a single clade, namely, eu‐D14 (Bythell‐Douglas et al., 2017; Kodama et al., 2023). A minor sister taxon to eu‐D14, unique to conifers but functionally uncharacterised, is known as DLK4. A much larger clade, DLK23, is sister to the combined DLK4 + eu‐D14 clade. DLK23 comprises divergent proteins from both gymnosperms and angiosperms, including DLK2 that is a transcriptional marker for KAI2 signalling (Bythell‐Douglas et al., 2017; Meng et al., 2021; Waters et al., 2012). Although the function of DLK23 proteins is uncertain and possibly varied, genetic and biochemical analyses imply that DLK23 homologues do not have an SL perception role (Bythell‐Douglas et al., 2017; Ramos‐Alvelo et al., 2024; Végh et al., 2017), and thus, the DLK4 + eu‐D14 and DLK23 clades likely represent functionally distinct groups of proteins. If so, the node at the base of the DLK4 + eu‐D14 clade would represent the earliest ancestor of the canonical SL receptor in seed plants and would be expected to exhibit properties typical for D14. However, given the relatively close relationship with DLK23 homologues and its inferred origins via gene duplication from a KAI2‐like ancestor, we might also expect a proto‐D14 to have broader or less specialised activity, especially in terms of bioactive substrates.

Ancestral sequence reconstruction (ASR) is a molecular evolution technique that relies on multiple sequence alignment and phylogenetic analysis to generate a sequence that may have existed in a common ancestor (Thornton, 2004). As an alternative to comparing many orthologous proteins, an ancestral sequence can be used to infer the function of a larger group. In plants, ASR has been used to understand the evolution of self‐incompatibility mechanisms in Brassicaceae through the generation of an ancestral S‐Locus Receptor Kinase, showing that the divergence of two allelic variants was asymmetrical: one variant resembles the specificity of the ancestor, while the other is neofunctionalised with novel ligand specificity (Chantreau et al., 2019). Another study used ASR to characterise the evolution of binding pockets in soluble epoxide hydrolases as a representative group (Bzówka et al., 2022). Protein engineering can also exploit features that typically arise as artefacts from ASR, such as increased thermostability (Gaucher et al., 2003, 2008; Gumulya et al., 2018; Wijma et al., 2013), and the ability to tolerate mutations that are destabilising but functionally useful (Risso et al., 2014). In addition to increased thermostability, or perhaps a result of it, ASR‐designed proteins tend to display elevated recombinant expression levels, making it easier to scale protein production for industrial applications (Lei et al., 2023). Another major desirable trait for protein engineering is the activity of an enzyme, both with respect to its turnover rate (Gumulya et al., 2018) and its specificity (Siddiq et al., 2017). ASR has proved useful for boosting enzyme activity against a broader range of substrates, thereby enhancing enzyme‐based industrial processes and biopharmaceutical development (Barruetabeña et al., 2019; Risso et al., 2013).

Here, we describe an ancestral reconstruction of D14 from seed plants. We found that the ancestral protein was structurally highly similar to extant D14 proteins, exhibited traits desirable for an engineered protein and behaved as a bona fide strigolactone receptor *in planta*. However, we did not find any evidence for wider substrate specificity relative to D14 from *A. thaliana*.

## RESULTS

### Inference of an ancestral D14 sequence

**Figure 1:**
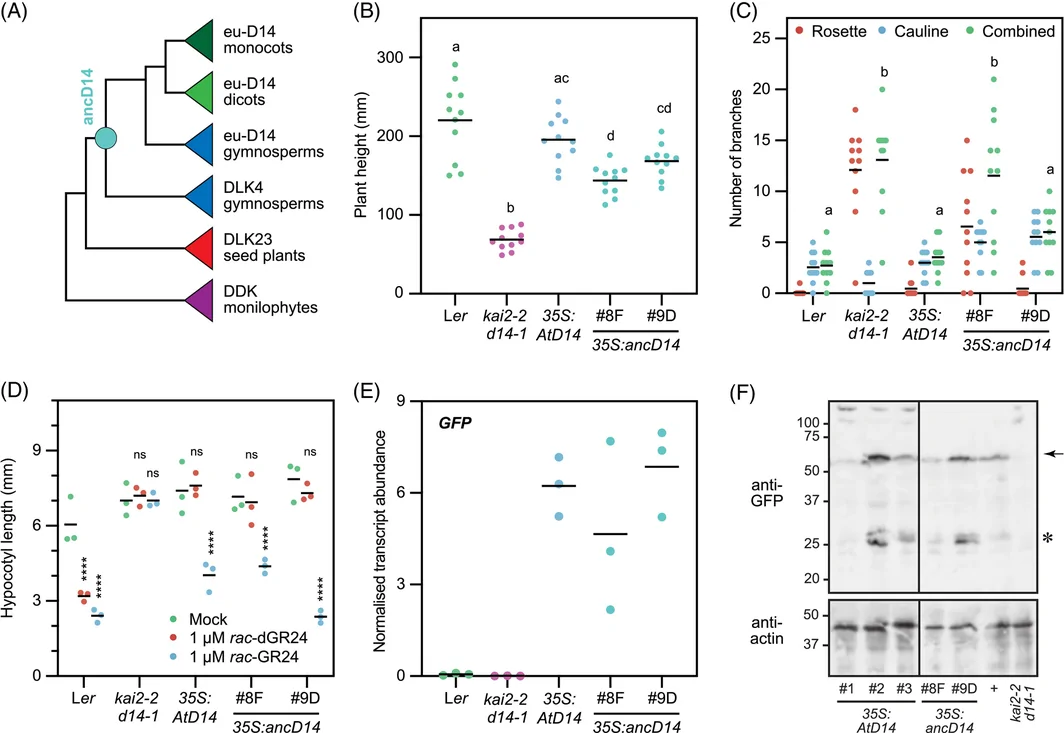
Phenotypes of *Arabidopsis thaliana* constitutively expressing ancD14. (A) ancD14 was designed to represent the ancestor of eu‐D14 and DLK4 sequences from seed plants. Simplified cladogram based on maximum likelihood phylogeny (Figure S2). (B) Scatter plot of plant height evaluated 18 days after the emergence of the primary inflorescence. Significantly different groups denoted by lowercase letters; *n* = 11. (C) Scatter plot of shoot branching observed in 80‐day‐old plants. Significantly different groups denoted by lowercase letters for the combined number of branches only; *n* = 11. (D) Suppression of hypocotyl elongation in relevant transgenic lines by synthetic strigolactone analogues. Each data point represents the mean of *n* = 3 independent replicate experiments, each comprising ≥20 seedlings per treatment. Results from individual replicate experiments are shown in Figure S5. Asterisks indicate comparisons with mock‐treated seedlings of the same genotype: ****P < 0.0001; ns, not significant (two‐way ANOVA with multiple pairwise comparisons). (E) Levels of *GFP‐AtD14* and *GFP‐ancD14* transcripts in stable transgenics, quantified by RT‐qPCR using primers anchored in the GFP coding region. Transcript abundance was normalised to the geometric mean of *CACS* and *TIP41L* transcripts. *n* = 3 biological replicates of 9‐day‐old seedlings; lines depict means. (F) Immunoblots targeting the GFP‐D14 fusion proteins (upper panel) and actin (lower). Gels were loaded with soluble protein extract from 9‐day‐old seedlings of two to three independent lines for each transgene. The blot was probed sequentially with anti‐GFP (Invitrogen A11122) and then anti‐actin (Sigma A0480) antibodies. The positive control (+) is an extract from Arabidopsis seedlings expressing GFP‐BdKAI2 fusion protein (Meng et al., 2021). The arrow depicts the anticipated GFP‐D14 fusion protein of ~60 kDa, and the asterisk indicates an assumed cleavage product of free GFP (~27 kDa). Line #3 of *35S:GFP‐AtD14* was used in this study and in panel A. Vertical line indicates where relevant samples from a larger blot were spliced together for presentation.

To generate a putative ancestral D14 sequence likely to function as a bona fide SL receptor representative of seed plants, a large dataset of DDK (D14/DLK2/KAI2) protein sequences was considered (Bythell‐Douglas et al., 2017). Sequences were compared within the DLK4 + eu‐D14 clade, which contains all true D14 sequences in seed plants (Figure 1A). Within this group, we looked at each core amino acid position identified in Bythell‐Douglas et al. (2017) and assessed whether the residue present at that position was highly conserved (found in 75% of sequences in the group), conserved (found in 50% of sequences in the group) or showed minority consensus (found in 30% of sequences in the group and the most common residue at that position). We then used this information to infer the most plausible residue at each position to derive ancD14, a single ancestral sequence representing the DLK4 + eu‐D14 clade (Figure S1). A maximum likelihood phylogeny of 86 protein sequences, encompassing ancD14, DLK4 + eu‐D14 from seed plants, and more distant DDK sequences from ferns and lycophytes, placed ancD14 in its expected position at the base of the strongly supported clade of DLK4 + eu‐D14 sequences (Figure S2). Notably, support values indicated that the position of ancD14 relative to the eu‐D14 and DLK4 subclades was ambiguous, consistent with it being representative of both groups.

To validate objectively our manual inference described above, we carried out an automated ancestral sequence reconstruction using FireProt‐ASR, which uses maximum likelihood to infer sequences at nodes of a phylogenetic tree (Musil et al., 2021). Supplying the pipeline with the same set of protein sequences (minus ancD14) generated a phylogeny with highly congruent topology to our maximum likelihood analysis, except for the relative placement of fern and lycophyte sequences (Figures S2 and S3). Node 88, which defined the seed plant DLK4 + eu‐D14 clade, represented a putative ancestral sequence that was identical to ancD14 at 234 of 262 (89%) comparable positions, with no gaps (Figure S4). Among the remaining 28 positions, 18 were conservative substitutions with similar biochemical properties that are not likely to result in a substantial change in protein folding or function. Among the remaining 10, eight were different again in AtD14, suggesting that they are not strongly conserved positions. Meanwhile, 10 positions had posterior probability <0.75, with several sites close to ambivalence (Figure S4). Thus, our manual inference method generated an ancestral sequence with a very close approximation to that generated by a non‐directed computational approach.

### Ancestral D14 complements the *d14* mutation of *A. thaliana*

To test if ancD14 can fulfil the role of its counterpart in *A. thaliana*, the ancD14 coding sequence was cloned into the plant binary vector pMDC43 (Curtis & Grossniklaus, 2003) to generate a protein fusion with GFP at the N terminus. Two independent transgenic lines homozygous for the *35S:GFP‐ancD14* transgene (hereafter *35S:ancD14*) were generated in the *kai2‐2 d14‐1* double mutant background, alongside an equivalent line expressing AtD14 as a control. Expression in the double mutant under a constitutive promoter allowed for an assessment of functional orthogonality, in case ancD14 should have any KAI2‐related activity. The functionalities of ancD14 and AtD14 were compared *in planta* with respect to plant height and shoot branching phenotypes.

The *kai2‐2 d14‐1* double mutant exhibited a dwarf stature compared to wild‐type L*er* throughout the reproductive phase of growth (Figure S5A). The *35S:AtD14* transgene largely recovered this dwarf phenotype, although there was a notable delay in flowering that presumably results from the *kai2‐2* mutation (Xu et al., 2022), because this phenotype was not affected by either of the transgenes (Figure S5B). Accordingly, plant height was evaluated 18 days after flowering initiated, rather than plant age, when phenotypic divergence was clear (Figure S5C). When compared to *35S:AtD14*, one of the two *35S:ancD14* lines was slightly shorter on average, but both were significantly taller than *kai2‐2 d14‐1* (Figure 1B). Therefore, with respect to plant height, we conclude that ancD14 offers near‐complete complementation of the *d14* phenotype in *A. thaliana*.

The *kai2‐2 d14‐1* double mutant displays increased shoot branching relative to L*er*, essentially due to additional rosette, rather than cauline, shoots. *35S:AtD14* was able to revert this mutant phenotype as expected (Figure 1C). The two lines expressing ancD14 exhibited different shoot branching phenotypes, with line #9D also restoring branching back to that of L*er*, but individuals of line #8F had very variable branching phenotypes, such that overall #8F was not significantly different from *kai2‐2 d14‐1* in this respect. From these data, we conclude that ancD14 can partially to fully recapitulate the *d14* phenotype in *A. thaliana*.

The transgenic Arabidopsis lines expressing AtD14 and ancD14 were also assayed for their ability to respond towards different ligands, by measuring the suppression of hypocotyl elongation (Figure 1D). Reintroduction of *AtD14* in the *kai2‐2 d14‐1* double mutant background allowed for responses to *rac*‐GR24, but not to *rac*‐dGR24. Both lines expressing ancD14 demonstrated the same pattern, with line #9D showing the greater response commensurate with its stronger expression of the transgene. These data confirm that ancD14 can function as a receptor for exogenous SL analogues in a similar manner to AtD14, including non‐canonical interactions with SMAX1 and/or SMXL2 that mediate hypocotyl growth responses to exogenous butenolides (Wang, Wang, et al., 2020). No evidence was found that ancD14 could compensate for loss of KL signalling through *KAI2* nor mediate responses to desmethyl *rac*‐dGR24, based on the elongated hypocotyl phenotype and the impaired transcription of the downstream marker *DLK2* (Figure S5E).

We suspected that partial complementation and differing phenotypes between the two independent lines expressing ancD14 could reflect differences in transgene expression level. RT‐qPCR revealed that line #8F, which exhibited weaker complementation of the observed phenotypes, had lower and less consistent transgene expression at the transcript level compared to the other transgenic lines (Figure 1E). These differences also resulted in weaker expression on the protein level (Figure 1F). These findings provide a plausible explanation for the incomplete and variable phenotypic complementation observed in line #8F.

### Ancestral D14 can bind and hydrolyse butenolides *in vitro*

To understand if ancD14 can act as a receptor and an enzyme towards SLs, recombinant ancD14 was expressed as a SUMO‐fusion protein and compared with AtD14 in several protein‐based assays. During these experiments, we found that the average yield of SUMO‐ancD14 was 5.1 mg l^−1^ of bacterial culture, more than twice that of SUMO‐AtD14 (Figure S6).

**Figure 2:**
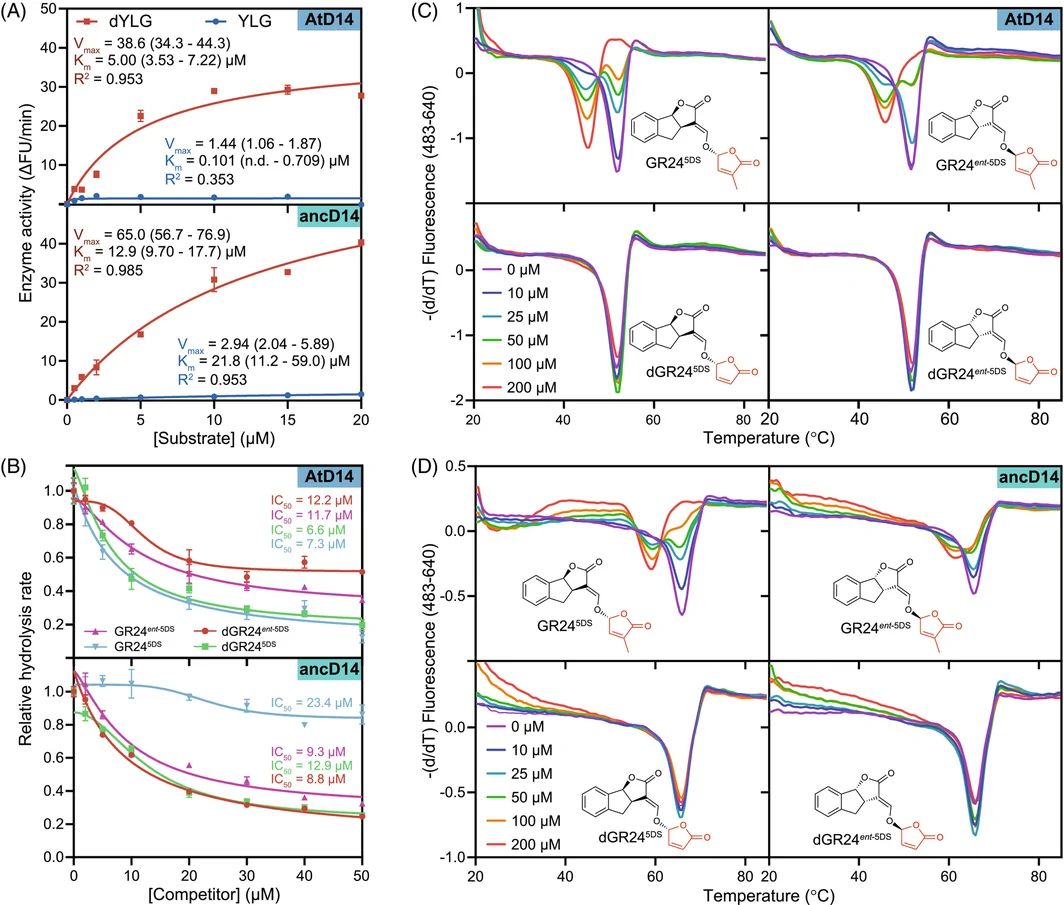
Ancestral D14 interacts with butenolides. (A) Hydrolysis of Yoshimulactone Green (YLG) and desmethyl Yoshimulactone Green (dYLG) by equivalent concentrations of AtD14 (top) and ancD14 (bottom). Data points are presented as means ± SE for *n* = 3 technical replicates; Michaelis–Menten values are provided with 95% CI estimates in brackets; n.d., not determinable. (B) Suppression of dYLG hydrolysis by GR24 and dGR24 compounds. Hydrolysis of 5 μM dYLG was competed with 0–50 μM GR24 or dGR24 enantiomers in the presence of 100 ng protein. Data points are presented as means ± SE for *n* = 3 technical replicates; estimates for IC_50_ values are provided. (C, D) Differential scanning fluorometry of AtD14 and ancD14 incubated with 0–200 μM of individual enantiomers of GR24 and desmethyl‐GR24 (dGR24). Data points are means of *n* = 4 technical replicates; insets show ligand structures.

D14 and its homologue KAI2 both show hydrolytic activity towards the profluorescent compound Yoshimulactone Green (YLG), with strongly enhanced activities towards desmethyl Yoshimulactone green (dYLG) (Tsuchiya et al., 2015; Yao et al., 2021; Yao, Mashiguchi, et al., 2018). We compared the activity of ancD14 with AtD14 to identify any changes in enzymatic activity or substrate promiscuity. We observed that ancD14 shows strong hydrolytic activity towards dYLG over YLG, in a similar fashion to AtD14 (Figure 2A). Notably, with concentrations of enzyme kept constant, ancD14 exhibited approximately double the maximal rate of enzymatic activity than AtD14 towards both substrates. However, *K*_m_ values differed considerably. Although the affinity of ancD14 for dYLG was just twofold lower than that of AtD14, it was some two orders of magnitude lower for YLG (Figure 2A). Thus, ancD14 is a more active enzyme than AtD14, but displayed much reduced affinity for the YLG substrate that most closely mimics natural strigolactones.

To investigate the substrate specificity of ancD14 further, we conducted hydrolysis competition assays with a constant concentration of dYLG, titrated against increasing concentrations of the constituent enantiomers of GR24 and dGR24. Inhibition of dYLG hydrolysis was used to infer a relative affinity of the enzyme for each enantiomer (Figure 2B). AtD14 activity was inhibited by all four enantiomers, but GR24^5DS^ and dGR24^5DS^ with SL‐type 2′*R*‐configured D‐rings were the most effective, as reported previously (Yao et al., 2021). Surprisingly, GR24^5DS^ was the only compound that failed to effectively compete with dYLG for hydrolysis by ancD14, suggesting that relative to AtD14, ancD14 does not show a strong preference for compounds with SL‐type stereochemistry.

To obtain further evidence of GR24 interacting with ancD14, differential scanning fluorimetry (DSF) was used to screen for thermal shifts indicative of ligand–receptor interactions. AtD14 was thermally destabilised by both GR24^5DS^ and GR24^*ent*‐5DS^ but not the dGR24 equivalents (Figure 2C), as reported previously (Waters et al., 2015; Yao et al., 2021). Consistent with hypocotyl elongation responses, the same ligand preference for GR24 over dGR24 was held by ancD14, albeit with a comparatively muted response to GR24^*ent*‐5DS^ (Figure 2D). In the absence of a ligand, ancD14 displayed a substantially elevated melting temperature (65.9°C) compared to that of AtD14 (51.7°C). Nevertheless, GR24^5DS^ lowered the melting temperature of AtD14 by 6.1°C (from 51.7 to 45.6°C) and of ancD14 by 6.6°C (from 65.9 to 59.3°C). Despite the large absolute differences in melting temperature, the qualitatively and quantitatively comparable ligand‐induced shifts in melting profile are consistent with a similar conformational change occurring in both proteins. These data indicate that, like AtD14 and other natural homologues (Yao et al., 2021), ancD14 retains a requirement for a ligand with a methyl‐substituted butenolide moiety to trigger this conformational change. Furthermore, the ligand‐induced conformational change in the isolated protein mirrors ligand bioactivity *in planta*.

### The structure of ancestral D14

**Figure 3:**
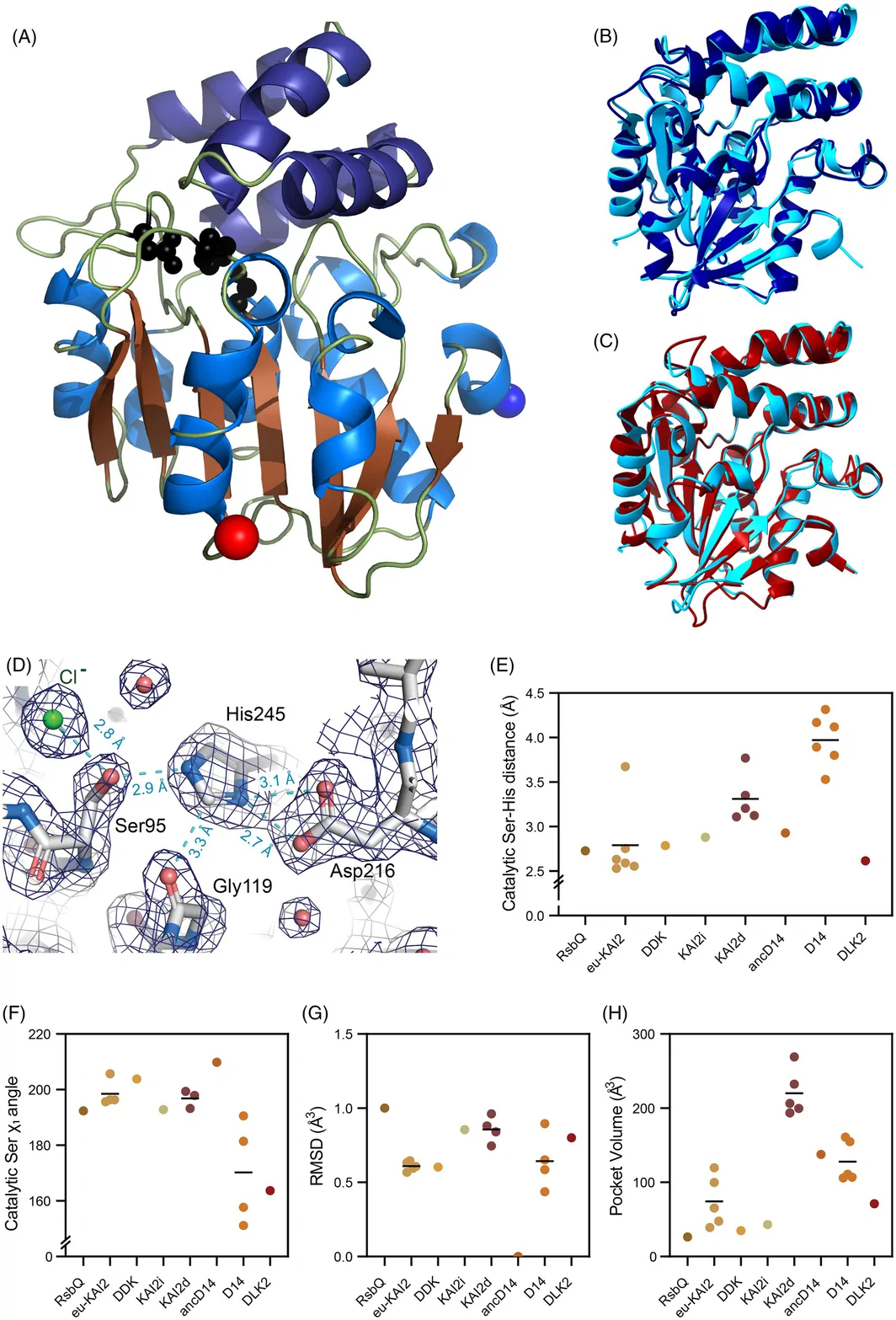
Crystal structure of ancestral D14 (PDB 7UKB). (A) Cartoon representation of ancD14 coloured by secondary structure (helices, blue; strands, brown). The N and C termini are represented by blue and red spheres, respectively. The catalytic triad is represented by black balls‐and‐sticks. (B) Structural alignment of ancD14 and AtD14 (4IH4); RMSD = 0.896 Å. (C) Structural alignment of ancD14 and AtKAI2 (4HRX); RMSD = 0.727 Å. (D) The active site of ancD14, in ball‐and‐stick representation. Blue mesh, 2mFo‐DFc electron density represented at 1.25 σ. Key interatomic distances are indicated by cyan dashed lines. (E–H) Comparison of catalytic serine‐histidine distance (E), catalytic serine χ_1_ angle (F), RMSD (compared to ancD14) (G) and pocket volume (H) between KAI2/D14‐type homologues. Where several structures are published of one homologue, the mean distance is indicated. Underlying data are provided in Table S1.

To investigate the structural characteristics of the ancestral D14 protein, the crystal structure of the recombinant protein was elucidated to a resolution of 1.95 Å (Figure 3; Table 1). As expected, the structure of ancD14 bears close similarity to other D14 family proteins and the α/β hydrolase family as a whole, based on a seven‐stranded mostly parallel β‐sheet flanked by α‐helices, with the active site located between the C‐terminal edge of the β‐sheet and a largely helical lid domain. All atom root‐mean‐square deviations range from 0.4 Å for *Petunia hybrida* D14, through other D14 and KAI2 variants to 1.0 Å for *Bacillus subtilis* RsbQ, in line with sequence similarity (Figure 3B,C,G; Table S1). The 1.95 Å resolution electron density of ancD14 is generally excellent, revealing clear conformations of side chains and well‐defined solvent molecules. Analysis of the active site geometry reveals features that place ancD14 apart from other D14 structures. For example, the distance between key atoms in the catalytic serine and histidine residues is substantially shorter than other D14 structures and more akin to that of DDK and eu‐KAI2 (Figure 3E). In addition, the χ_1_ dihedral angle of serine 95 is at the extreme of the distribution of χ_1_ angles in the whole family (Figure 3F), with other D14 proteins lying at the other extreme. However, the overall pocket volume was estimated to be typical of that of a eu‐D14 protein (Figure 3G). To explore the significance of these observations, close inspection of all available structures of homologues revealed that the active site geometry is influenced by the presence of solvent molecules or ions adjacent to the catalytic serine. In the case of ancD14, a chloride ion is observed at this position, while in the cluster of structures with similar χ_1_ angles a water, chloride or magnesium ion is modelled. Thus, it is possible that the specific detail of active site geometry of α/β hydrolases may be influenced by the crystallisation conditions as much as by homology. Nevertheless, the structure of ancD14 is functionally intact and displays the conformation and relevant interactions expected for a competent enzyme.

**Table 1:** X‐ray data collection and refinement statistics

Data collection^a^	
Space group	P4_3_2_1_2
*a*, *b*, *c* (Å)	58.79, 58.79, 146.07
α, β, γ (°)	90, 90, 90
Resolution range (Å)	48.69–1.95 (1.99–1.95)^b^
Total no. of reflections	511 574 (34144)
No. of unique reflections	19 665 (1333)
Completeness (%)	99.9 (98.0)
Redundancy	26.0 (25.6)
⟨*I*/σ(*I*)⟩	11.0 (1.0)
*R*_p.i.m._	0.047 (0.617)
*CC*_1/2_	0.999 (0.595)
Wilson *B*‐factor (Å^2^)	25.5
Refinement	
Resolution range (Å)	45.797–1.946 (2.016–1.946)^b^
Completeness (%)	99.8 (98.2)
No. of reflections, working set	18 578 (1794)
No. of reflections, test set	989 (88)
Final *R*_work_	0.1842 (0.2691)
Final *R*_free_	0.2325 (0.3255)
No. of non‐H atoms	
Protein	2055
Ion	1
Water	237
Total	2293
R.M.S. deviations	
Bonds (Å)	0.0016
Angles (°)	0.46
Average B‐factors (Å^2^)	
Protein	30.54
Ion	29.28
Water	36.69
Ramachandran plot	
Most favoured (%)	97.32
Allowed (%)	2.68
Outliers (%)	0.00

^a^ Data were collected from a single crystal.

^b^ Values for the outer shell are given in parentheses.

Seeking a structural basis for the increased thermostability of ancD14, we used Arpeggio (Jubb et al., 2017) to investigate the distribution of various inter‐residue contacts across members of the family. We found several of the classes of contact were highly variable among five different structures of the same protein, suggesting they would be insufficiently robust to explain small differences between related proteins (Table S2). However, when considering those classes that were stable, we observed that hydrophobic contacts were positively correlated with T_m_, at least when comparing ancD14 with native mesophilic proteins for which we have melting point data (i.e. AtD14 and OsD14; Yao et al., 2021). This tentative finding fits with the general observation of thermostability observed in ancestrally reconstructed proteins being due to the sum of many small factors. Ancestral protein thermostability reflects the same mechanism of stabilisation as consensus protein thermostability (Trudeau et al., 2016), which itself has been attributed to the fact that taking the most common residue at any position will eliminate less frequent variants that are slightly detrimental to structural stability (Williams et al., 2006).

### Strigolactone signalling via ancD14 does not require an intact catalytic triad

Previous research has established that substitution of the catalytic aspartic acid residue of AtD14 with alanine (D218A) does not prevent perception of strigolactones *in vivo*, that is, the AtD14^D218A^ variant can complement the *d14‐1* mutant phenotype (Seto et al., 2019). This result is surprising because AtD14^D218A^ does not support hydrolysis activity, while analogous mutations of the catalytic serine (S97A) and histidine (H247A) residues block both signalling and ligand hydrolysis in Arabidopsis (Seto et al., 2019) and rice (Hu et al., 2017). These observations imply that the catalytic serine and histidine residues are important for ligand binding, but that ligand hydrolysis *per se* is not a requirement for SL signalling. However, formally this result could be idiosyncratic of the AtD14 protein. As a representative of all seed plant D14 proteins, ancD14 offers a powerful opportunity to address the generality of the hydrolysis requirement.

**Figure 4:**
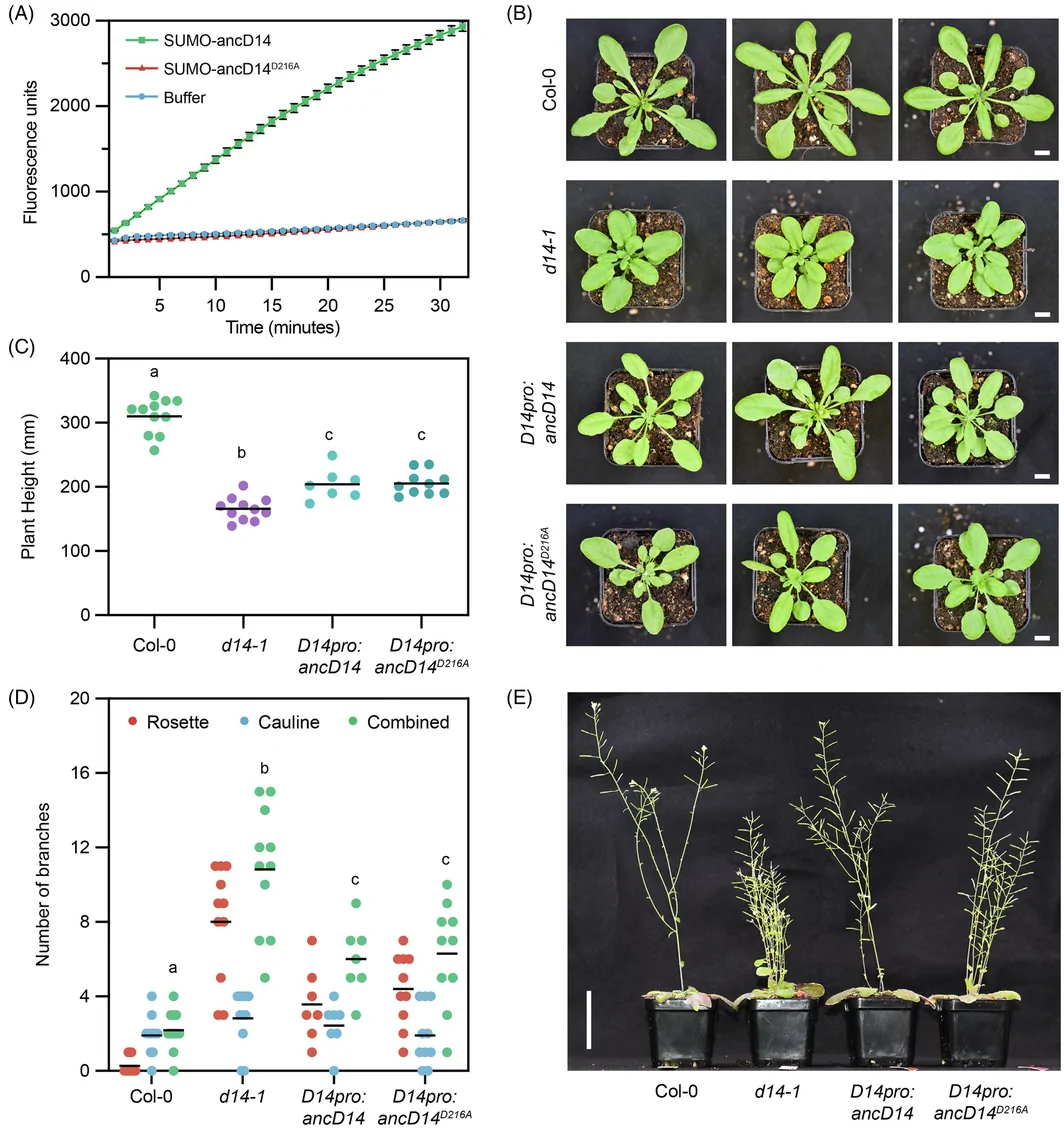
The catalytic aspartic acid residue is not essential for ancD14 signalling. (A) Hydrolysis activity of SUMO‐tagged ancD14 and ancD14^D216A^ against 20 μM dYLG. Data points are mean ± SE for *n* = 4 technical replicates. (B) Rosette phenotypes at 32 days post‐germination. For *D14pro:ancD14* and *D14pro:ancD14* ^*D216A*^, each plant is an independent transgenic individual (T_1_ generation). (C) Scatter plot of plant height evaluated 18 days after the emergence of the primary inflorescence. Significantly different groups denoted by lowercase letters; *n* = 11 individuals, except for *D14pro:ancD14* (*n* = 10) and *D14pro:ancD14* ^*D216A*^ (*n* = 7), where each data point represents an independent transgenic individual (T_1_ generation). (D) Scatter plot of shoot branching observed in 46‐day‐old plants. Significantly different groups denoted by lowercase letters for the combined number of branches only; sample sizes as per (C). (E) Photographs of representative plants for each genotype at 54 days of age.

We first confirmed that the equivalent D216A mutation abolished the hydrolysis activity of ancD14 against dYLG (Figure 4A). To test functionality *in planta*, GFP‐ancD14 and GFP‐ancD14^D216A^ variants were expressed under the native Arabidopsis *D14* promoter (*AtD14pro*) in the *d14‐1* single mutant. This choice of background allowed for more accurate recapitulation of native D14 activity and also served as a counterpoint for previous constructs driven by the *35S* promoter and in the double *kai2 d14* mutant (Figure 1). Multiple independent transgenic events were considered by assessing phenotypic rescue in the T_1_ generation. We found that ancD14^D216A^ complemented the *d14‐1* phenotype equivalently to the original ancD14 in terms of leaf and rosette growth, plant height and shoot branching (Figure 4B–E; Figure S7). Neither transgene was able to fully restore the mutant phenotype to that of wild‐type Col‐0, especially in terms of plant height. This trend was also found with the *35S*‐based transgenes, suggesting that it is a property of ancD14 itself, rather than that of the expression domain or strength. Nevertheless, ancD14^D216A^ was indistinguishable from ancD14 with respect to all phenotypes examined, suggesting that ligand hydrolysis is generally dispensable for SL receptor function.

## DISCUSSION

### Ancestral D14 can functionally substitute for AtD14

Combined biochemical and biological data indicate that ancD14 can fulfil the same functions as AtD14. Transgenic expression in either the *kai2‐2 d14‐1* double or *d14‐1* single mutant background showed that ancD14 can replicate the SL signalling roles of AtD14 in *A. thaliana* with respect to leaf morphology, plant height and shoot branching. Besides endogenous SLs, ancD14 and AtD14 perceived exogenous GR24 equivalently, as judged by hypocotyl elongation responses. The preference of ancD14 towards butenolide ligands *in vitro* was essentially typical for D14 proteins, with DSF responses mimicking those of AtD14. This functional conservation spans a remarkable evolutionary timeframe: seed‐bearing plants emerged in the fossil record during the late Devonian, about 360 Ma (Bateman et al., 2026; Linkies et al., 2010), while an ancestral polyploidy event occurred in the common ancestor of extant seed plants around 320 Ma (Jiao et al., 2011). But likewise, given the evolutionary distance and inference involved, it is not surprising that ancD14 displayed some performance penalty relative to modern equivalents.

First, the phenotypic complementation by ancD14 was incomplete: plant height of *d14‐1* could not be restored fully to that of wild type, regardless of expression strength, while shoot branching levels approached that of wild type only when ancD14 was strongly expressed. This intermediate phenotypic rescue was discernible using two different promoters and across multiple transgenic lines. The consistent partial complementation suggests that the reduced activity may reflect intrinsic properties of ancD14, although a direct comparison with GFP‐AtD14 expressed from the D14 promoter construct was not performed.

Second, ancD14 showed relatively poor affinity for the compounds we would expect to represent endogenous chemical regulators of shoot branching, that is, a methyl‐substituted butenolide ring in a 2′*R* stereoconfiguration. In particular, the K_m_ for YLG was very high compared to AtD14, even though its maximum catalytic rate with dYLG outperformed that of AtD14. Furthermore, the hydrolysis activity of ancD14 was inhibited poorly by GR24^5DS^, suggesting that this compound is unable to compete strongly for the active site. This result was counterintuitive, considering that GR24^5DS^ resembles the 2′*R*‐configuration of endogenous SLs most closely and that GR24^5DS^ was active in DSF. It is formally possible that the GR24^5DS^ was simply consumed by ancD14 extremely rapidly, meaning that less competitor was present to inhibit dYLG hydrolysis. However, this is unlikely because GR24^5DS^ was a very effective competitor for AtD14, and GR24^*ent*‐5DS^ also competed well with ancD14. Additionally, the poor catalytic activity of ancD14 towards YLG does not favour an argument for a very fast catalytic rate towards GR24^5DS^. Our structural analysis implies that ancD14 is somewhat an outlier with regard to the Ser‐His distance and the Ser95 χ_1_ dihedral angle, when compared to native D14 proteins. The unusual active‐site geometry may be consistent with altered catalytic or ligand‐binding properties, although we have not established a direct causal relationship.

A secondary aim of this study was to ask if an ancestral D14 protein might have a shifted ligand preference, perhaps showing signalling activity with 4′‐desmethyl butenolides. We found no evidence that these butenolides are bioactive via ancD14, and therefore, the strong rate of dYLG hydrolysis by ancD14 and its capacity to bind dGR24 do not imply a broader ligand range for signalling. Instead, and like AtD14 (Yao et al., 2021), we infer that these desmethyl butenolide compounds act only as hydrolysis substrates for ancD14, without having the capacity to activate the receptor function by triggering a conformational change. Thus, we conclude that the absolute substrate preference has not changed, but that the selectivity of ancD14 towards endogenous SLs may be more relaxed relative to AtD14 and other natural receptors. It is possible that the unusual active‐site geometry may contribute to the altered ligand‐binding and catalytic properties of ancD14, but this interpretation remains tentative because the observed conformation may also be influenced by crystallisation conditions or the local crystal environment. Regardless of this uncertainty, the compromised performance of ancD14 as an SL receptor may result from a combination of effects: reduced affinity for endogenous SLs, but also because of suboptimal interactions with other SL signalling partners such as MAX2 or SMXL/D53 with which native D14 proteins will have co‐evolved. In addition, the stability of ancD14 may have changed relative to native proteins, which could have corresponding effects on signalling dynamics (Sánchez Martín‐Fontecha et al., 2024). Screening a greater variety of natural SLs and analogues, both *in vitro* and *in vivo*, would help to assess these uncertainties more comprehensively.

### Ancestral D14 exhibits desirable traits for biotechnology

As ancD14 behaves in a comparable manner to AtD14, it serves as a useful tool to exploit its advantageous traits, such as heightened thermostability, elevated expression yield in *Escherichia coli* and increased enzymatic activity, while preserving the essential functions of a D14 protein. The enhanced expression yield of ancD14 represents an opportunity to scale up D14 protein expression and facilitate the use of D14 in high‐throughput mutant screens. The drastically enhanced thermostability also renders ancD14 an ideal platform for introducing further destabilising mutations that might not otherwise be tolerated. These beneficial attributes broaden the utility of the protein in applications such as a biosensor, or as a functional standard with improved usability and storage characteristics.

We did not test the activity of ancD14 in plants beyond *A. thaliana*, so it is not known for certain whether ancD14 can mediate cereal traits such as tillering. However, D14 is highly conserved and appears to be interchangeable within angiosperms; for example, D14 from *O. sativa* can fully complement the mutant phenotype when introduced to *A. thaliana* (Yao, Wang, et al., 2018). An ASR of D14 specific to angiosperms (rather than the more distant seed plant ancestor) would be helpful in establishing this principle more comprehensively. Recently, it was discovered that SL signalling in rice is enhanced by the N‐terminal domain (NTD) of D14. Under conditions of low nitrogen availability, phosphorylation of serine residues in the NTD increases the protein's stability and prolongs signalling lifetime (Hu et al., 2024). However, this NTD is only found in monocot D14 homologues, and likewise, the regulatory mechanism that acts via the NTD may be monocot‐specific as well. Conceivably, the improved stability of ancD14, or an angiosperm‐focussed variant, could be exploited to allow for a similar trait to be introduced to other crop species. Conversely, the enhanced enzymatic activity of ancD14 could serve to create an SL‐decreased environment; for example, by mutating the residues associated with MAX2 interaction to abolish signalling and expressing it under a conditional promoter, perhaps SL signalling could be ‘knocked down’ in a context‐dependent manner.

### Strigolactone signalling is tolerant of structural variation at the ligand binding site

Overall, the structure of ancD14 is similar to other D14 structures published previously. The most notable point of difference between ancD14 and extant homologues lies in the configuration of the active site. Specifically, ancD14 possesses a compact catalytic triad where the χ_1_ angle of the serine residue and the distance between the serine and histidine residues are more characteristic of a KAI2‐type protein. Although this observation is consistent with an early strigolactone receptor emerging from a KAI2‐like ancestor, these structural features did not result in any substantive changes in ligand preference *in vitro* or functional performance *in planta*, suggesting that these features are not critical for ligand selectivity. Likewise, divergent KAI2 proteins such as those from the moss *Physcomitrium patens* or the lycophyte *Selaginella moellendorffii* plausibly serve as SL receptors because non‐seed plants often respond to SL‐like compounds but lack D14‐like sequences or predicted structures (Bythell‐Douglas et al., 2017, Lopez‐Obando et al., 2021, Waters et al., 2015). It is also noteworthy that the substrate preferences are easily altered: for example, as few as three substitutions in conserved residues can confer strigolactone response upon a KAI2 protein (Arellano‐Saab et al., 2021). Therefore, if the ability to perceive SLs does not require a highly specific structural conformation at the active site, perhaps the ability to perceive SLs is instead more dictated by the capacity to recruit cognate signalling partners, specifically MAX2 and/or SMXL proteins.

### Insights and limitations of ASR for understanding D14 evolution

It is challenging to draw definitive conclusions about protein evolution from a single ancestral reconstruction alone. ASR relies heavily on accurate phylogenetic trees and sequence alignments, and different models of evolution can reveal different ancestral states. At 89% site identity, there were some deviations between our manually inferred ancD14 sequence and that generated by FireProt‐ASR, reflecting expected ambiguities in the ancestral state. Although the remaining 11% of sites could have contributed to the experimental outcomes we report, they were probably inconsequential. First, it should be noted that the goal of ASR is to infer ancestral function, not a definitive ancestral sequence. However, more importantly, systematic investigations have demonstrated that ancestral proteins are highly robust to uncertainty, even when scores of ambiguous amino acids are altered. Although enzymatic parameters, for example, can fluctuate between different ASRs, gross functional differences—such as changing an enzyme into a receptor or changing the motif that a DNA binding domain recognises—are not (Eick et al., 2016). Our results with ancD14 are consistent with such general conclusions about ASR: we did not change D14 into KAI2 or even make it sensitive to desmethyl butenolides, but we did alter its substrate binding properties and thermal stability, without affecting its overall capacity to serve as a hormone receptor.

The ability of AtD14^D218A^ to support signalling without SL cleavage (Seto et al., 2019) was reproduced by the equivalent ancD14^D216A^, which similarly lost the capacity to hydrolyse dYLG. This outcome suggests that the uncoupling of signalling from ligand hydrolysis may represent an evolutionarily conserved feature of seed‐plant D14 proteins. It is implausible that this apparently superfluous ligand hydrolysis activity would be maintained for so long without an adaptive basis; presumably, localised and rapid removal of the active signal is an intrinsic part of a precision signalling mechanism, because AtD14^D218A^ mutants are hypersensitive to exogenous GR24 (Seto et al., 2019). It remains an interesting open question whether ligand hydrolysis is also dispensable for KAI2 proteins; in our experience, the corresponding D218A mutation is not tolerated by KAI2, at least from *A. thaliana*.

It has been previously proposed that SL signalling may have more relaxed structural requirements than KAI2‐mediated signalling (Bythell‐Douglas et al., 2017), in which case attributes such as the more compact active site architecture, and/or the potential reduced affinity for SLs of ancD14 that was observed *in vitro*, may reflect a transitional and relatively unrefined phase in the evolution of SL signalling. Arguably, later evolution might have selected for improved ligand affinity, potentially conferring greater sensitivity and spatial resolution upon SL response. Otherwise, we hypothesise that the functional specialisation of D14 as an SL‐specific receptor accompanied the emergence of the seed plants and may even have assisted in their subsequent radiation. The ability of ancD14 to perceive exogenous GR24 and endogenous SLs *in planta* provides evidence that interactions between D14 and MAX2 or SMXL‐family proteins emerged at a comparable time and are conserved throughout seed plants. Such *in vivo* functional testing remains relatively rare for ASR studies, and these insights encourage further adoption of this methodology to answer evolutionary questions.

## METHODS

### Generation of ancestral sequences

The ancestral D14 sequence was derived manually based on sequence alignment. For 221 of 264 positions, there was clear conservation of amino acid usage within the DLK4 + eu‐D14 clade, and thus a straightforward decision for those positions in the ancestral sequence. For the remaining 43 positions, there was no clear consensus within the DLK4 + eu‐D14 clade, so we instead considered consensus from a wider group of proteins, comprising 85 non‐redundant sequences in all: eudicot D14, monocot D14, basal angiosperm D14, gymnosperm D14, gymnosperm DLK4, gymnosperm DLK4A, gymnosperm DLK4B, fern DDK and lycophyte DDK. Based on the distribution of residues across these groups, it was usually possible to establish a likely ancestral residue. For instance, for position 43, angiosperm D14 proteins have a consensus for tyrosine (Y), but both gymnosperm D14 and gymnosperm DLK4 proteins have a consensus for phenylalanine (F). Thus, angiosperm 43Y is derived, and 43F is likely ancestral. For position 128, DLK4 sequences have a consensus for glycine (G) while eu‐D14 has a consensus for aspartic acid (D); however, fern DDK proteins (the sister group to DLK4 + eu‐D14) have a consensus for aspartic acid, implying that the ancestral residue is 128D. In this way, we could assign a plausible ancestral residue for a further 39 positions. For the remaining four positions, there was no clear pattern of residues across the clades, and we selected the most frequent amino acid as the consensus across those groups.

Automated ancestral reconstruction was performed using FireProt^ASR^ v 2.0 (Musil et al., 2021) with AtD14 specified as the query. Posterior probabilities were tabulated in Excel.

### Phylogenetic analysis

Amino acid sequences were aligned using MAFFT v7.490 (algorithm: Auto, scoring matrix BLOSUM62, gap open penalty 1.53, offset value 0.123). No sequence trimming was performed. Phylogenies were created using PhyML v3.3.20180621 implemented in Geneious Prime, using the WAG substitution model with 100 bootstraps, optimising for topology and branch length.

### Molecular cloning

A synthetic DNA fragment encoding ancD14 was codon optimised for *E. coli* (GenScript GenSmart) and cloned into pDONR207 (ThermoFisher Scientific). For recombinant expression, ancD14 was re‐amplified with ~25‐bp overhangs and assembled into the linearised pE‐SUMO Amp backbone via Hot Fusion assembly (Fu et al., 2014). A similarly codon‐optimised version of AtD14 was assembled directly into pE‐SUMO‐Amp. Site‐directed mutagenesis to generate ancD14^D216A^ was performed by inverse PCR.

To generate *35S:GFP* binary vectors, ancD14 (*E. coli* codon optimised) and AtD14 (native) coding sequences in pDONR207 were recombined with the pMDC43 destination vector (Curtis & Grossniklaus, 2003). To generate *D14pro:GFP* binary vectors, the *35S* promoter was excised from pMDC43 with *Hind*III and *Kpn*I, and a fragment corresponding to the *AtD14* promoter and 5′ UTR (Waters et al., 2015) was amplified by PCR and combined with the digested vector by Hot Fusion assembly. Subsequently, this modified destination vector was recombined with pDONR207‐based entry vectors carrying *ancD14* or *ancD14* ^*D216A*^ coding sequences. Recombinant plasmids were verified via colony PCR, restriction digest and Sanger sequencing. Oligonucleotides are listed in Table S3.

### Recombinant expression and purification

Expression vectors were transformed into Rosetta *E. coli* (Novagen) and selected on carbenicillin (100 μg ml^−1^) and chloramphenicol (34 μg ml^−1^). Three colonies were used to inoculate 5 ml LB plus antibiotics, grown at 30°C/180 rpm overnight as a starter culture and diluted 1:1000 into 450 ml LB containing carbenicillin (100 μg ml^−1^). Cultures were grown at 30°C/180 rpm to OD_600_ >0.5, chilled to 16°C and induced with 0.1 mM IPTG. Cultures were incubated at 16°C/180 rpm for a further 16 h and cells collected via centrifugation (5000× ***g***, 15 min). Cell pellets were stored at −70°C.

Proteins were purified at 4°C via affinity chromatography with 20 ml gravity columns. Cells were lysed by resuspending pellets in 12.5 ml lysis buffer (20 mM HEPES, 150 mM NaCl, 10% (v/v) glycerol, 20 mM imidazole, 1× BugBuster [Merck], pH 7.5). Lysates were complexed with 2 ml (settled volume) Ni‐NTA resin (BioRad) for the purpose of protein assays, or TALON cobalt resin (TaKaRa) for the purpose of crystallisation, for 1 hour with gentle rotation. Columns were washed with 20 ml wash buffer (20 mM HEPES pH 7.5, 150 mM NaCl, 10% (v/v) glycerol, 20 mM imidazole, pH 7.5) twice. Proteins were eluted in 10 ml elution buffer (20 mM HEPES, 150 mM NaCl, 10% glycerol (v/v), 200 mM imidazole, pH 7.5). Imidazole was diluted at least 1000‐fold via buffer exchange (with 20 mM HEPES, 150 mM NaCl, 10% (v/v) glycerol, pH 7.5) in 10 kDa centrifugal filter units (Amicon Ultra‐15 10 000 NMWL). For crystallisation purposes, proteins were dialysed overnight in the presence of SUMO protease UlpI to remove the 6xHIS‐SUMO leader sequence and purified by passing through cobalt affinity resin and collecting flow‐through only. Proteins were then concentrated by ultrafiltration using centrifugal filter units. Purity was assessed by SDS‐PAGE, and protein concentration was estimated via UV spectrometry or by Bradford assay (Bio‐Rad) to directly compare protein yields (Figure S6).

### Protein crystallisation

AncD14 crystals were grown by vapour diffusion in sitting drop format. AncD14 protein sample at 10 mg ml^−1^ was mixed with reservoir solution in a 1:1 ratio to produce a total drop volume of 0.4 μl and equilibrated at 293 K over a reservoir containing 60 μl of 25% (w/v) PEG 1500. Small square bipyramidal crystals grew after ~3–4 weeks to a maximum size of ~100 μm (Figure S6).

### X‐ray diffraction data collection

Single crystals were cryoprotected by transferring into Paratone‐N (Hampton Research) before being frozen in liquid nitrogen. Diffraction data were collected at the Australian Synchrotron MX1 beamline (Cowieson et al., 2015) at 100 K using an X‐ray energy of 13 keV. Data on a single crystal were collected continuously for 36 sec using an EIGER2 X 9M direct single‐photon counting detector (Dectris) while rotating the crystal through 360° such that the final dataset contained 3600 frames, each containing data from rotation through a *φ* angle of 0.1°.

### Data processing and refinement

Data were indexed using *XDS* (Kabsch, 2010), and scaled and merged in spacegroup *P* 4_3_2_1_2 using *AIMLESS* (Evans & Murshudov, 2013). The structure was solved by molecular replacement using *phaserMR* (McCoy et al., 2007) with the X‐ray crystal structure of *A. thaliana* KAI2 (PDB ID: 4HTA) (Bythell‐Douglas et al., 2013) as the search model. The model was initially rebuilt in *Coot* (Emsley et al., 2010) to correct for sequence differences between KAI2 and ancD14 before subsequent rounds of automated and manual refinement using *phenix.refine* (Afonine et al., 2012) and *Coot*, respectively, using data to a resolution of 1.95 Å. Refinement of anisotropic B‐factors was restricted to protein atoms contained in three translation–libration–screw (TLS) groups defined automatically using *phenix.refine*. Data collection and refinement statistics are listed in Table 1.

### Structure analysis

Geometric measurements and molecular graphics were generated using PyMOL (Schrodinger, 2015) and UCSF ChimeraX (Meng et al., 2023). Root‐mean‐square deviations calculated with the ‘align’ function of PyMol used all atoms. Dihedral (χ) angles were measured using ‘get_dihedral’ in PyMOL. Distances between the catalytic Ser and His residues (at the OG and NE2 atoms, respectively) were measured using the ‘Distance’ function of ChimeraX. Pocket volumes were estimated using CASTpFold (Ye et al., 2024).

### Chemical synthesis

Synthesis and enantiomeric separation of GR24, dGR24, YLG and dYLG were carried out as previously described (Mangnus et al., 1992; Scaffidi et al., 2014; Tsuchiya et al., 2015; Yao et al., 2021; Yao, Mashiguchi, et al., 2018).

### Hydrolysis assays

Hydrolysis assays were performed in technical triplicates using 96‐well black assay plates as described previously (Yao et al., 2021). Blank‐subtracted fluorescence values (from wells without protein) were analysed in GraphPad Prism, using nonlinear regression with the ‘Michaelis–Menten’ model. The initial hydrolysis rate was defined as the change in blank‐subtracted fluorescence units over the linear phase of the reaction.

### Hydrolysis competition assays

Competition assays were performed as described previously (Yao et al., 2021), with dYLG at a fixed concentration of 5 μM. Enantiomers of GR24 or dGR24 were included at 0–50 μM. Data analysis was performed in GraphPad Prism, using nonlinear regression with the ‘[Inhibitor] vs. response – Variable slope (four parameters)’ model.

### Differential scanning fluorometry

DSF assays were performed in technical quadruplicate using sealed 384‐well white assay plates and with a LightCycler 480 instrument (Roche), as described previously (Yao et al., 2021).

### Complementation of *A. thaliana*

Binary vectors were transformed into competent GV3101 *Agrobacterium tumefaciens*, with transformants verified via colony PCR. Homozygous *kai2‐2 d14‐1* double mutants in *A. thaliana* in Landsberg *erecta* background (Waters et al., 2015) or *d14‐1* single mutants in Columbia‐0 background (Waters et al., 2012) were transformed via floral dip. Primary transformants were selected on 0.5× MS agar supplemented with 20 μg ml^−1^ hygromycin B. T_2_ offspring were screened for 3:1 segregation of resistance to hygromycin B and further propagated to the T_3_ generation, in which homozygotes were selected that demonstrated 100% resistance to hygromycin B. Homozygous transformed lines were verified by PCR genotyping, RT‐qPCR and immunoblotting. T_1_ generation transformants (Figure 4) were confirmed by PCR genotyping.

### Immunoblotting

Total protein was extracted from 9‐day‐old *A. thaliana* seedlings, which were in liquid nitrogen and homogenised with two Zirconex beads in a mixer mill. Proteins were extracted in 150 μl buffer (50 mM Tris pH 7.5, 150 mM NaCl, 10% glycerol, 0.1% Tween‐20, 1 mM dithiothreitol, 1 mM phenylmethylsulfonyl fluoride, 1× Complete Protease Inhibitor Cocktail [Roche]) to 100 mg homogenised seedling tissue and removing cell debris via centrifugation at 4°C (12 000× ***g***, 10 min; followed by 15 000× ***g***, 10 min). Protein concentration was estimated by NanoDrop A280, using the BSA standard, and 80 μg protein extract was separated via 12% SDS‐PAGE. Proteins were transferred to a polyvinylidene fluoride membrane using a Trans‐Blot Turbo (Bio‐Rad).

Immunoblots were blocked with 1× TBS‐T (20 mM Tris‐HCl, 150 mM NaCl, 0.1% Tween‐20, pH 7.5) supplemented with 2% BSA at room temperature for 2 h. Antibodies were prepared by dilution to 10 ml with 1× TBS‐T with 0.2% BSA and incubated with the immunoblot at 4°C with gentle rocking for at least 2 h. Immunoblots were washed five times (5 min per wash) with 1× TBS‐T between each antibody incubation. GFP was probed using rabbit anti‐GFP (Invitrogen A11122), followed by goat anti‐rabbit IgG HRP (ThermoFisher 31460) and imaged using Clarity Western ECL substrate (Bio‐Rad). The immunoblot was then re‐blocked as above for a further 2 h and probed for actin mouse anti‐actin (Sigma‐Aldrich A0480) followed by goat anti‐mouse IgG AP (Pierce 31321), imaged using ImmunStar AP substrate (Bio‐Rad). Chemiluminescence was imaged with an ImageQuantRT ECL system (GE Healthcare).

### Transcript analysis

Total RNA was extracted from 9‐day‐old *A. thaliana* seedlings using the Spectrum Plant Total RNA Kit (Sigma‐Aldrich), following ‘Protocol A’ as described by the manufacturer. RNA was then treated with TURBO DNase (Life Technologies) and used in a cDNA synthesis reaction using the LunaScript RT SuperMix Kit (NEB). RT‐qPCR was performed using threefold‐diluted cDNA with Luna Universal qPCR Master Mix (NEB). Oligonucleotides are listed in Table S3.

### Hypocotyl elongation assays

Hypocotyl elongation assays were carried out as described previously (Sun et al., 2020). Three independent experimental replicates (Figure S5) were combined and statistical analysis performed on the complete dataset (*n* = 3).

### Measurement of shoot branching and plant height

Seedlings were germinated from surface‐sterilised seed on 0.5× MS agar, stratified in darkness at 4°C for 72 h, then grown under constant white light (~120 μmol m^−2^ sec^−1^) for 1 week at 21°C. For mature growth, 11 seedlings of each genotype were moved to a mixture of peat, perlite and vermiculite (6:1:1) and grown under long‐day conditions (16/8 h and 22/16°C day/night cycle, ~120 μmol m^−2^ sec^−1^ broad spectrum white light). Shoot branching, plant height (defined as inflorescence height above rosette) and flowering time were recorded daily.

### Statistical analysis

Data were analysed with one‐ or two‐way ANOVA, implemented in GraphPad Prism v10, with correction as noted in figure legends.

## CONFLICT OF INTEREST

The authors declare no competing interests.

## Supporting information


**Figure S1.** The ancestral‐reconstructed D14 sequence aligned to D14 and DLK4 sequences from seed plants. Sequences were aligned by MAFFT v7.49 and are grouped by similarity to each other. Gymnosperms, blue; monocots, green; dicots, fuschia; basal angiosperms, black; ancD14, orange.

**Figure S2.** Maximum likelihood phylogeny of vascular plant D14 homologues alongside ancD14. Sequence labels and clades are coloured according to major plant groups. The node representing the DLK4 + euD14 clade of seed plants is highlighted with a red circle. Node labels represent bootstrap support.

**Figure S3.** Phylogeny generated during automated ancestral sequence reconstruction by FireProt‐ASR. Sequence labels and clades are coloured according to major plant groups. Node 88, representing the DLK4 + euD14 clade of seed plants, is highlighted with a red circle. Note that node labels represent putative ancestors and sequences as generated by FireProt‐ASR and do not indicate support values for the tree topology.

**Figure S4.** Sequence comparison of ancD14 and closest equivalent ancestral sequence from FireProt‐ASR. (A) Alignment of ancD14, the FireProt‐ASR ancestral sequence node_88 and AtD14 from *Arabidopsis thaliana*. Arrowhead indicates start of conservation analysis at position 5, as the N terminus is typically highly variable and the calculated node_88 ancestor lacks an initial methionine. AtD14 is included for comparison, but the analysis of conservative and ambivalent alternatives only applies to the pairwise differences between ancD14 and node_88. For orientation purposes, the catalytic residues are highlighted with red asterisks. (B) Posterior probabilities associated with the 28 residues that vary between ancD14 and node_88. Positions in node_88 with posterior probabilities less than 0.75 are highlighted in red and are the same as those identified with black/yellow symbols in (A). Alternative residues and their corresponding probabilities are shown in brackets.

**Figure S5.** Detailed growth characterisation of *35S:GFP‐ancD14* transgenic lines. (A) Plant height over time defined as days post‐germination. (B) Scatter plot of flowering time for each genotype, lines represent the mean for that genotype, lowercase letters represent significantly different groups. (C) Plant height over time, defined as days post‐initiation of flowering. Data are representative of mean ± SE for *n* = 11 biological replicates. (D) Individual experimental replicates of hypocotyl elongation assay (relating to Figure 1D). Each panel represents a separate experiment in which *n* ≥ 23 seedlings per genotype–treatment combination were measured. Data presented as a box plot, with outliers (exceeding 1.5 × interquartile range deviation from the median) shown as individual points. (E) Relative expression level of *DLK2* transcripts in 9‐day‐old seedlings quantified by RT‐qPCR. Transcript abundance was normalised to the geometric mean of *CACS* and *TIP41L* transcripts; *n* = 3 biological replicates; lines depict means.

**Figure S6.** Heterologous expression of AtD14 and ancD14 in *Escherichia coli*. SUMO‐fusion proteins were expressed in triplicate 50‐ml cultures and purified from 10 ml samples using a crude magnetic bead chromatography method. (A) Estimates of yield were made through Bradford assay interpolated from a standard curve in which known concentrations of SUMO‐ancD14 were used (left Y‐axis), and by densitometry (relative to a non‐specific reference band; right Y axis) from the SDS‐PAGE gel (B). (C) Diffraction‐quality single crystals of ancD14. Scale bar: 100 μm.

**Figure S7.** Growth characterisation of Arabidopsis *D14pro:ancD14* transgenics. Data are representative of mean ± SE for *n* = 11 biological replicates, except for *D14:ancD14* (*n* = 10) and *D14:ancD14* ^*D216A*^ (*n* = 7), where each data point represents an independent first‐generation transformant. (A) Plant height over time defined as days post‐germination. (B) Plant height over time, defined as days post‐initiation of flowering.

**Data S1.** Protein sequences used in phylogenetic and FireProt‐ASR analyses.

**Data S2.** Nucleotide coding sequences for ancD14 and ancD14^D216A^.

**Table S1.** List of structures used for comparisons.

**Table S2.** Analysis of inter‐residue contacts with Arpeggio.

**Table S3.** Oligonucleotides used in this study.

## Data Availability

All data that support the findings presented in this article are available within the manuscript and the accompanying Supporting Information. Structural data for ancD14 are available via the Protein Data Bank under accession 7UKB.
