# Supplementary material for: Structural and functional characterisation of a reconstructed ancestral strigolactone receptor
Source: Plant J. 2026 Sep 14;127(5):e71113. doi: 10.1111/tpj.71113 (PMC13575467; doi:10.1111/tpj.71113)
Supplement: Supplementary file 1 [file TPJ-127-0-s001.pdf]

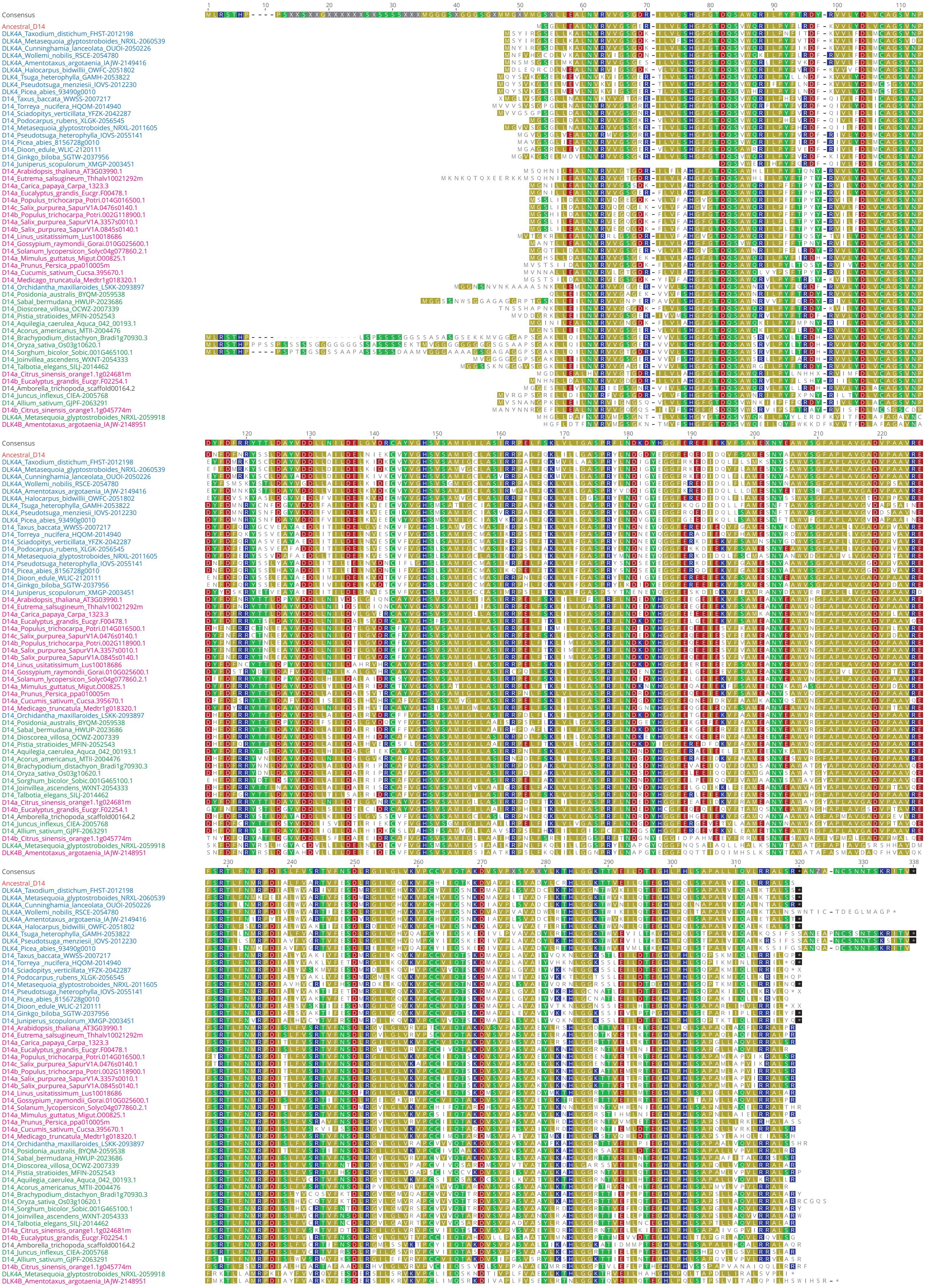

Figure S1 The ancestral-reconstructed D14 sequence aligned to D14 and DLK4 sequences from seed plants.
Sequences were aligned by MAFFT v7.49 and are grouped by similarity to each other. Gymnosperms, blue; monocots, green; dicots, fuschia; basal angiosperms, black; ancD14, orange.
