# Supplementary material for: Structural and functional characterisation of a reconstructed ancestral strigolactone receptor
Source: Plant J. 2026 Sep 14;127(5):e71113. doi: 10.1111/tpj.71113 (PMC13575467; doi:10.1111/tpj.71113)
Supplement: Supplementary file 2 [file TPJ-127-0-s002.pdf]

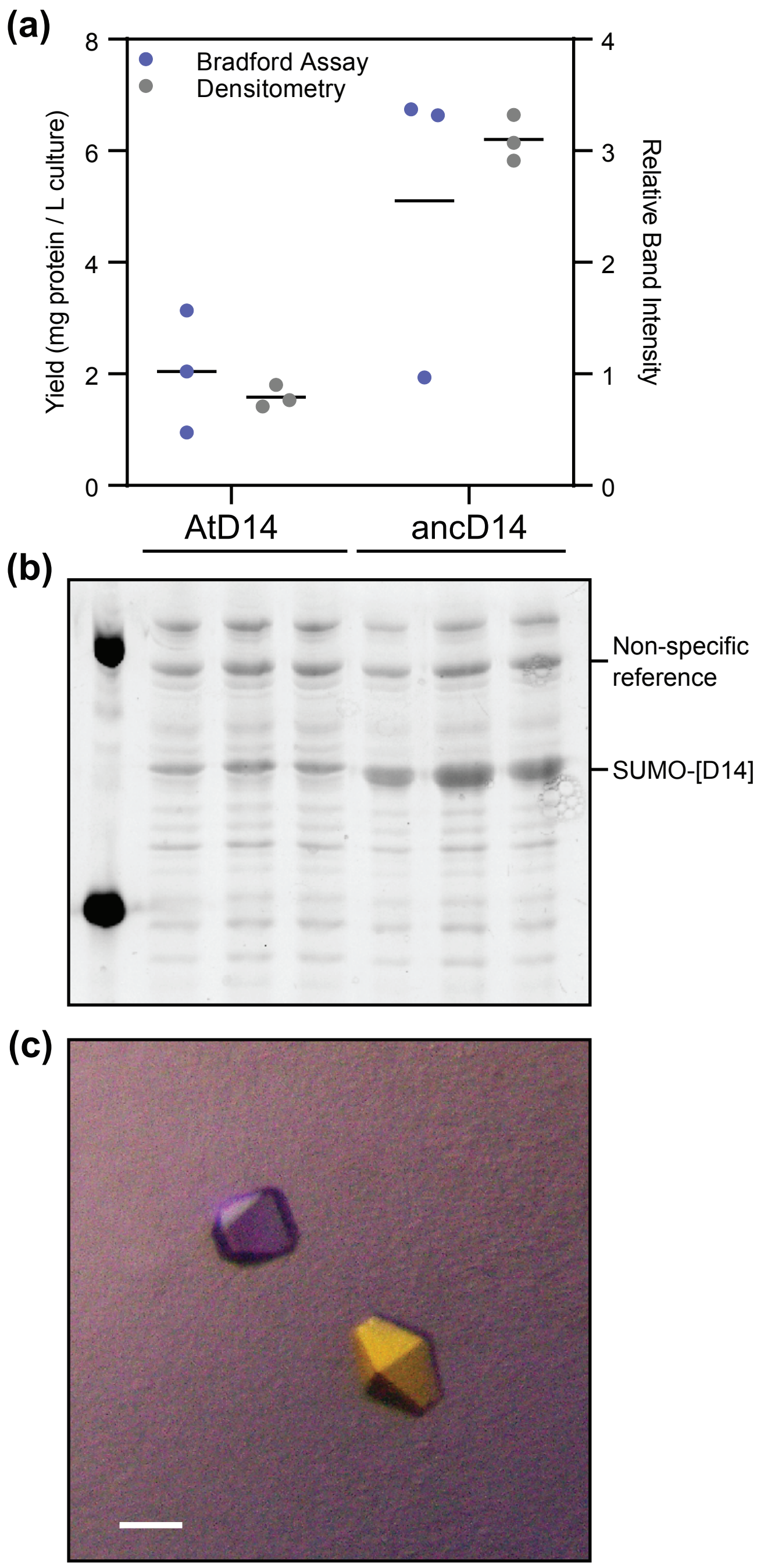

Figure S6 Heterologous expression of AtD14 and ancD14 in *E. coli*.
SUMO-fusion proteins were expressed in triplicate 50-mL cultures, and purified from 10 mL samples using a crude magnetic bead chromatography method. (**a**) Estimates of yield were made through Bradford assay interpolated from a standard curve in which known concentrations of SUMO-ancD14 were used (left Y axis), and by densitometry (relative to a non-specific reference band; right Y axis) from the SDS-PAGE gel (**b**). (**c**) Diffraction-quality single crystals of ancD14. Scale bar: 100 µm.
