# Supplementary material for: Structural and functional characterisation of a reconstructed ancestral strigolactone receptor
Source: Plant J. 2026 Sep 14;127(5):e71113. doi: 10.1111/tpj.71113 (PMC13575467; doi:10.1111/tpj.71113)
Supplement: Supplementary file 3 [file TPJ-127-0-s003.pdf]

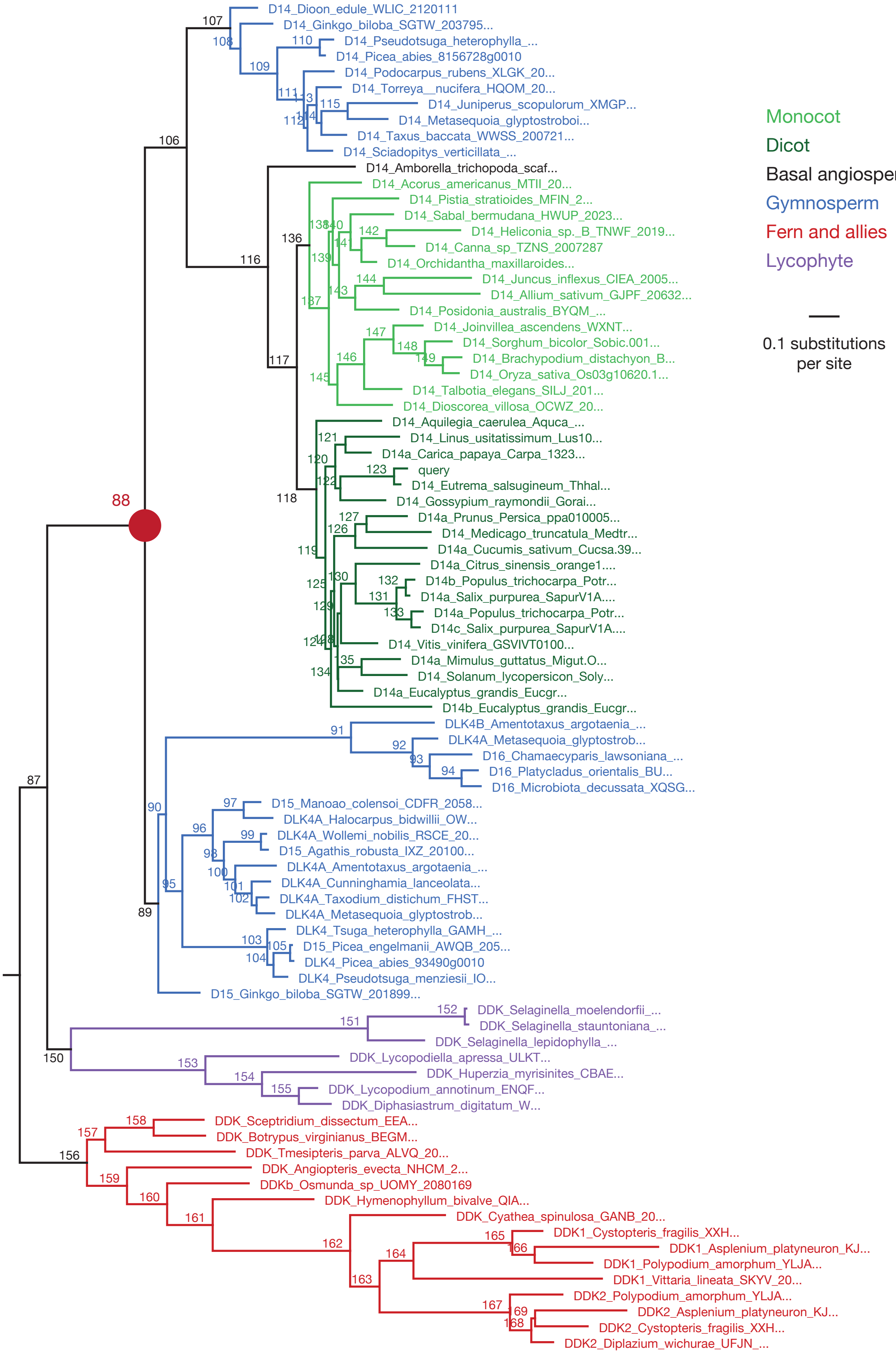

Figure S3 Phylogeny generated during automated ancestral sequence reconstruction by FireProt-ASR
Sequence labels and clades are coloured according to major plant groups. Node 88, representing the DLK4+euD14 clade of seed plants, is highlighted with a red circle. Note that node labels represent putative ancestors and sequences as generated by FireProt-ASR, and do not indicate support values for the tree topology.
