# Supplementary material for: Structural and functional characterisation of a reconstructed ancestral strigolactone receptor
Source: Plant J. 2026 Sep 14;127(5):e71113. doi: 10.1111/tpj.71113 (PMC13575467; doi:10.1111/tpj.71113)
Supplement: Supplementary file 4 [file TPJ-127-0-s006.pdf]

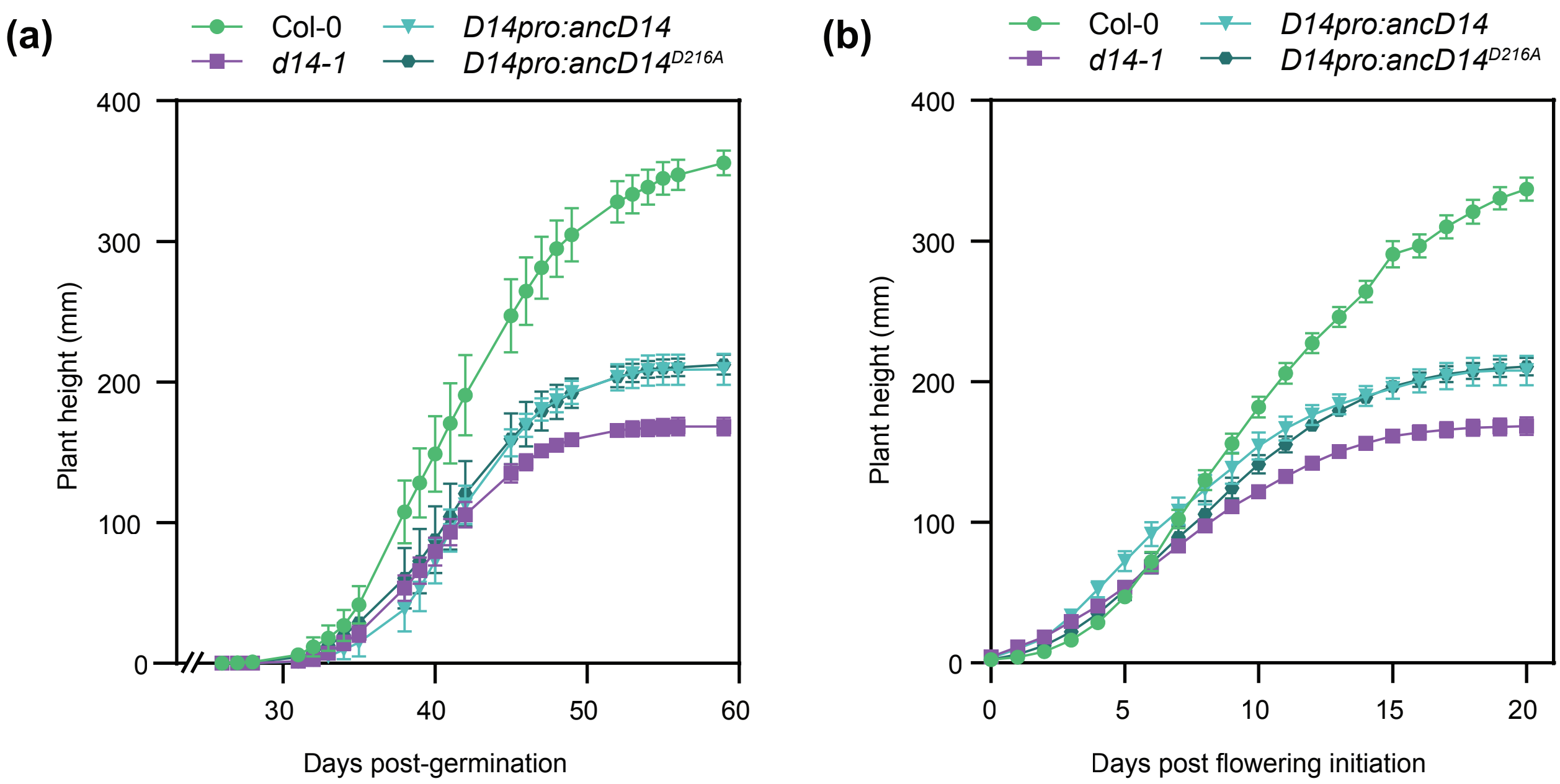

Figure S7 Growth characterisation of Arabidopsis *D14pro:ancD14* transgenics.
Data are representative of mean ± SE for n = 11 biological replicates, except for *D14pro:ancD14* (n = 10) and *D14pro:ancD14*^{D216A} (n = 7), where each data point represents an independent first-generation transformant. (**a**) Plant height over time defined as days post-germination. (**b**) Plant height over time, defined as days post-initiation of flowering.
