# Supplementary material for: Structural and functional characterisation of a reconstructed ancestral strigolactone receptor
Source: Plant J. 2026 Sep 14;127(5):e71113. doi: 10.1111/tpj.71113 (PMC13575467; doi:10.1111/tpj.71113)
Supplement: Supplementary file 5 [file TPJ-127-0-s007.pdf]

(a)

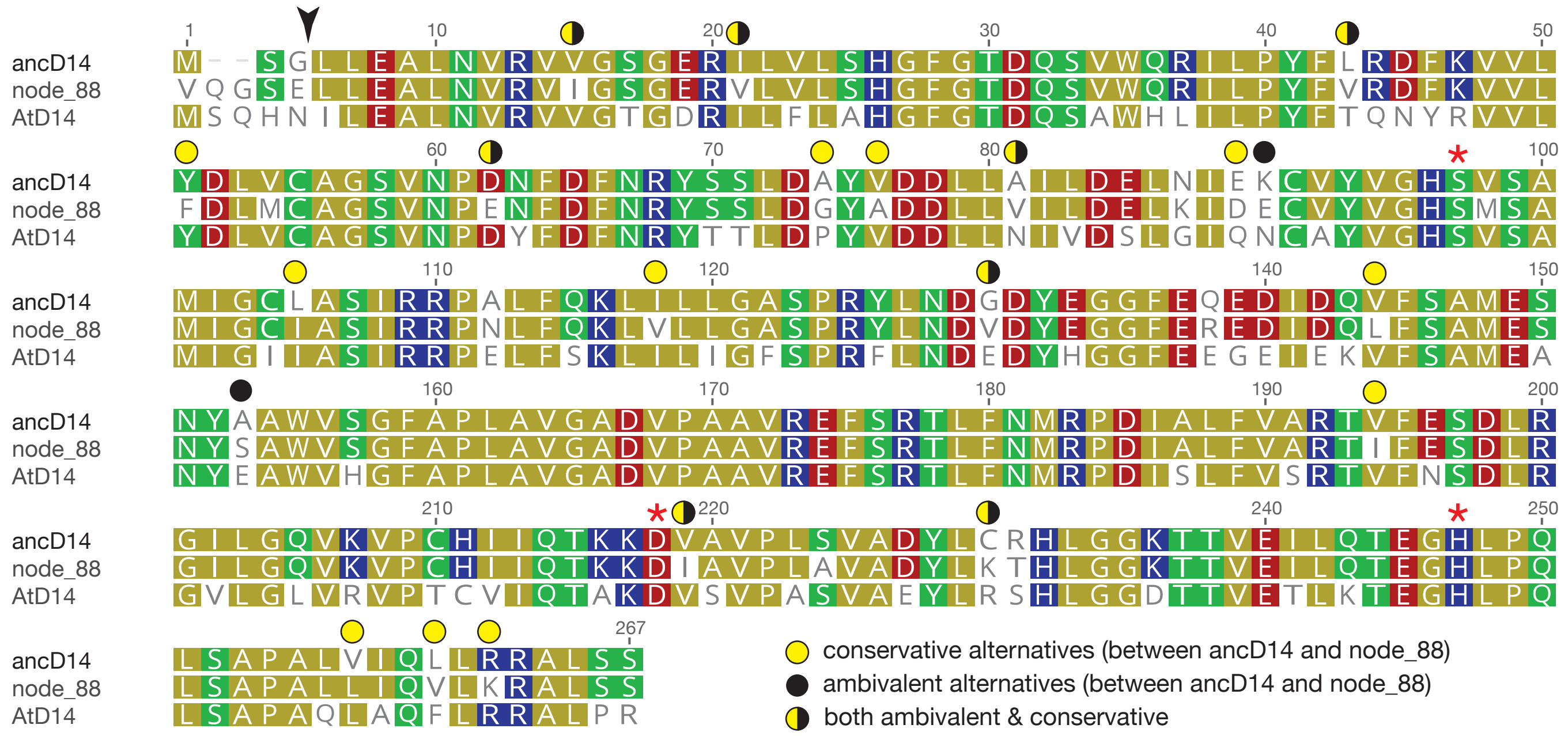

(b)

Alignment position	ancD14 residue	node_88 residue	Posterior probability	Alignment position	ancD14 residue	node_88 residue	Posterior probability
15	V	I (V)	0.55 (0.41)	112	A	N	0.9
21	I	V (I)	0.50 (0.48)	118	I	V	0.99
43	L	V (I) (L)	0.47 (0.31) (0.18)	130	G	V (I)	0.74 (0.17)
51	Y	F	0.99	138	Q	R	0.97
54	V	M	0.86	144	V	L	0.98
62	D	E (D)	0.53 (0.46)	153	A	S (E) (A)	0.63 (0.11) (0.08)
74	A	G	0.87	194	V	I	0.96
76	V	A	0.99	219	V	I (M) (V)	0.7 (0.14) (0.07)
81	A	V (A) (T)	0.38 (0.26) (0.22)	224	S	A	0.98
87	N	K	0.94	230	C	K (R)	0.74 (0.25)
89	E	D	0.91	231	R	T	0.77
90	K	E (K)	0.41 (0.37)	257	V	L	0.85
98	V	M	0.96	260	L	V	0.79
105	L	I	0.94	262	R	K	0.86

Figure S4 Sequence comparison of ancD14 and closest equivalent ancestral sequence from FireProt-ASR
(a) Alignment of ancD14, the FireProt-ASR ancestral sequence node_88, and AtD14 from *Arabidopsis thaliana*. Arrowhead indicates start of conservation analysis at position 5, as the N-terminus is typically highly variable and the calculated node_88 ancestor lacks an initial methionine. AtD14 is included for comparison, but the analysis of conervative and ambivalent alternatives only applies to the pairwise differences between ancD14 and node_88. For orientation purposes, the catalytic residues are highlighted with red asterisks.
(b) Posterior probabilities associated with the 28 residues that vary between ancD14 and node_88. Positions in node_88 with posterior probabilities less than 0.75 are highlighted in red, and are the same as those identified with black/yellow symbols in (a). Alternative residues and their corresponding probabilities are shown in brackets.
