# Supplementary material for: Structural and functional characterisation of a reconstructed ancestral strigolactone receptor
Source: Plant J. 2026 Sep 14;127(5):e71113. doi: 10.1111/tpj.71113 (PMC13575467; doi:10.1111/tpj.71113)
Supplement: Supplementary file 6 [file TPJ-127-0-s009.pdf]

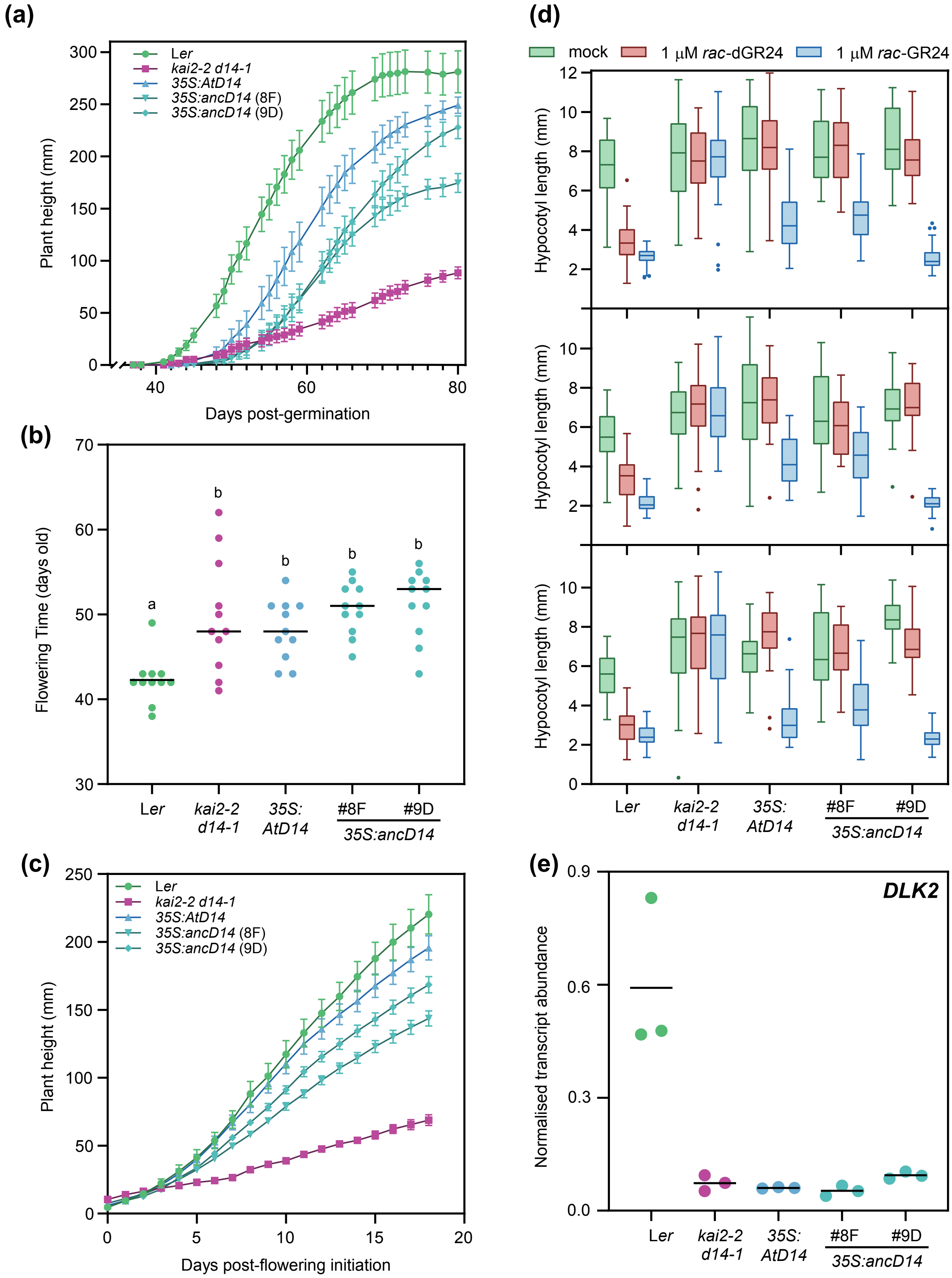

Figure S5 Detailed growth characterisation of *35S:GFP-ancD14* transgenic lines.
(**a**) Plant height over time defined as days post-germination. (**b**) Scatter plot of flowering time for each genotype, lines represent the mean for that genotype, lower case letters represent significantly different groups. (**c**) Plant height over time, defined as days post–initiation of flowering. Data are representative of mean ± SE for n = 11 biological replicates. (**d**) Individual experimental replicates for hypocotyl elongation assay (relating to Figure 1d) . Each panel represents a separate experiment in which n ≥ 23 seedlings per genotype-treatment combination were measured. Data presented as a box plot, with outliers (exceeding 1.5 × interquartile range deviation from the median) shown as individual points. (**e**) Relative expression level of *DLK2* transcripts in 9-day-old seedlings quantified by RT-qPCR. Transcript abundance was normalised to the geometric mean of *CACS* and *TIP41L* transcripts; n = 3 biological replicates; lines depict means.
