# Supplementary material for: Structural and functional characterisation of a reconstructed ancestral strigolactone receptor
Source: Plant J. 2026 Sep 14;127(5):e71113. doi: 10.1111/tpj.71113 (PMC13575467; doi:10.1111/tpj.71113)
Supplement: Supplementary file 7 [file TPJ-127-0-s011.pdf]

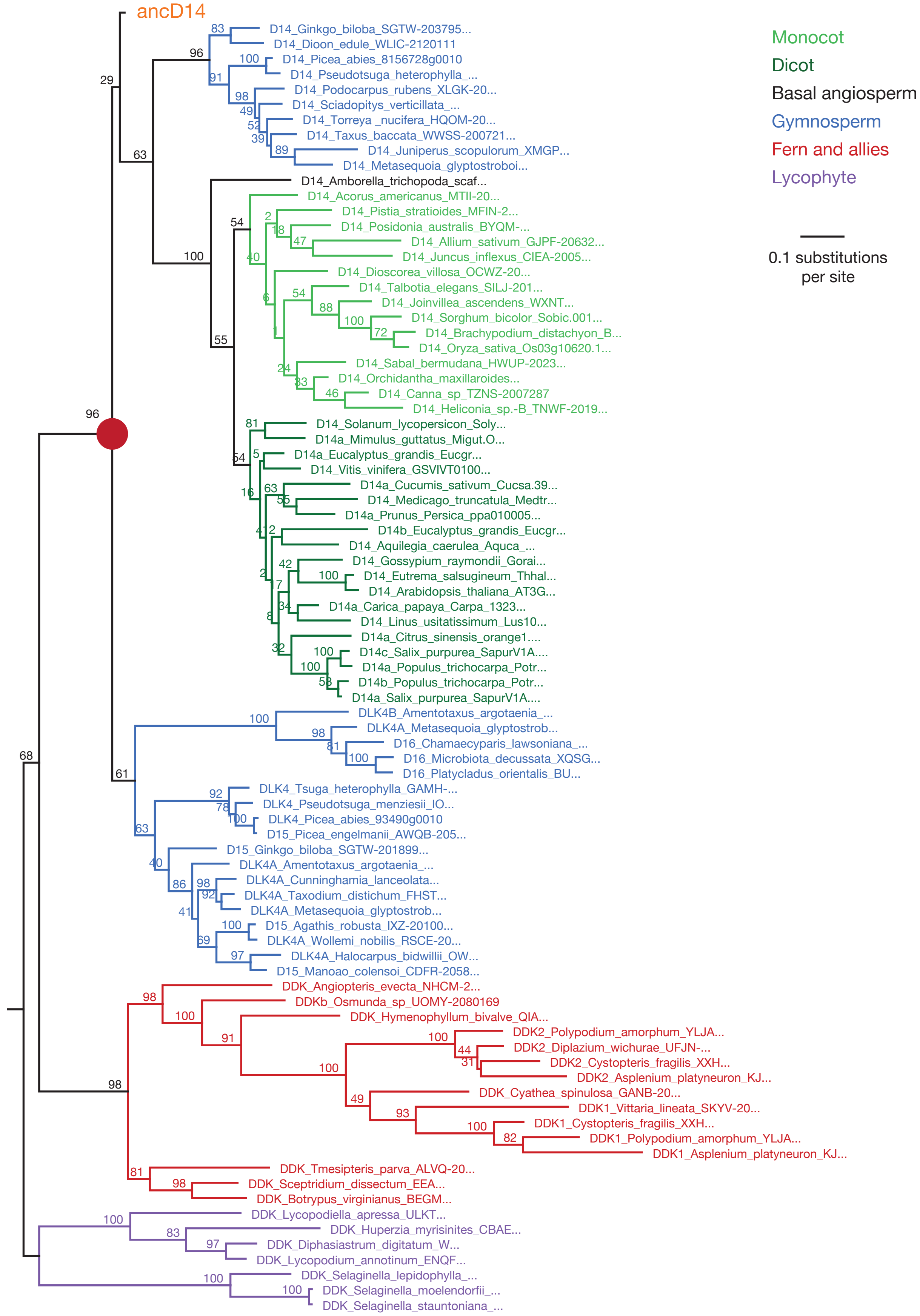

Figure S2 Maximum likelihood phylogeny of vascular plant D14 homologues alongside ancD14
Sequence labels and clades are coloured according to major plant groups. The node representing the DLK4+euD14 clade of seed plants is highlighted with a red circle. Node labels represent bootstrap support.
